# Design of Experiments (DoE)‐Optimized Polymeric Oxytocin Nanoparticles for Enhanced Nose‐to‐Brain Delivery

**DOI:** 10.1002/smll.202511603

**Published:** 2025-12-19

**Authors:** Naveed Ahmad, Shunping Han, Rifka Utami, Rafal Baker, Dina Helal, Zhuoni Li, Mark Tricklebank, Yannis Paloyelis, Julie Wang, Marija M. Petrinovic, Sukhi Bansal, Khuloud T. Al‐Jamal

**Affiliations:** ^1^ Institute of Pharmaceutical Science King's College London Franklin‐Wilkins Building, 150 Stamford Street London SE1 9NH UK; ^2^ Faculty of Pharmacy Hasanuddin University Makassar 90245 Indonesia; ^3^ Department of Pharmaceutics and Industrial Pharmacy Faculty of Pharmacy Ain Shams University Monazzamet El Wehda El Afrikeya Street, Abbasseya Cairo Egypt; ^4^ Department of Forensic and Neurodevelopmental Sciences Institute of Psychiatry Psychology and Neuroscience King's College London 16 De Crespigny Park London SE5 8AF UK; ^5^ Department of Neuroimaging, Institute of Psychiatry Psychology and Neuroscience King's College London De Crespigny Park London SE5 8AF UK; ^6^ Medical Research Council Centre for Neurodevelopmental Disorders King's College London New Hunt's House London SE1 1UL UK; ^7^ Department of Pharmacology and Pharmacy Li Ka Shing Faculty of Medicine The University of Hong Kong Hong Kong Special Administrative Region China

**Keywords:** artificial intelligence, autism, autoradiography, encapsulation, peptide, PLGA, radiolabeling

## Abstract

Oxytocin (OT) is a promising candidate for regulating social behavior in autism spectrum disorder (ASD). However, its inconsistent efficacy can be attributed to the lack of an efficient delivery system that selectively target the brain without inducing peripheral side effects following intranasal (IN) administration. In this study, OT is encapsulated within an FDA‐approved poly (lactic‐co‐glycolic acid) (PLGA) nanoparticles (OT‐NP) to improve nose‐to‐brain (NTB) delivery. A PEGylated version (OT‐NP‐PEG) is developed to improve nasal mucosal diffusion. Optimization using a design of experiments (DoE) approach produced nanoparticles with hydrodynamic diameters of ≈93–116 nm, polydispersity index ≈0.20, zeta potential –21 to –33 mV, and drug loading ≈2.8–3.5% (w/w). The stable OT‐NP‐PEG showed sustained release (>42% and 58% at 24 and 72 h) and greater diffusion through simulated nasal mucus. [^14^C] OT is synthesized with chemical and radiochemical yields of 74% and 53%, respectively. Following IN administration in mice, [^14^C] OT‐NP‐PEG demonstrated rapid brain uptake, particularly in the olfactory bulb and frontal cortex, with reduced blood and liver exposure compared with free [^14^C] OT. Finally, IN OT‐NP‐PEG significantly increased self‐grooming frequency in mice, indicating maintained bioactivity and behavioral effects. Overall, OT‐NP‐PEG offers a rationally designed nanoplatform for brain‐targeted OT delivery in ASD and other neuropsychiatric disorders.

## Graphical abstract

Intranasal (IN) delivery of PEGylated PLGA nanoparticles (NPs) loaded with oxytocin (OT) (OT‐NP‐PEG) is investigated as a strategy to enhance OT transport to the brain for treating autism and other neuropsychiatric disorders. The optimized NP formulation demonstrates appropriate physicochemical characteristics, improved stability, and enhanced mucosal diffusion. In vivo organ‐biodistribution study of [^14^C] OT‐NP‐PEG shows increased brain uptake following IN administration and preserved bioactivity in a self‐grooming behavioral assay, highlighting its therapeutic potential for OT‐associated brain disorders.

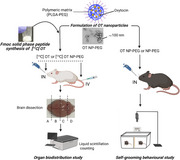

## 1 Introduction

Oxytocin (OT) is a neuropeptide hormone largely synthesized in the hypothalamic supraoptic and paraventricular nuclei and is known to have both neuronal, neuroendocrine and endocrine effects. OT is transported to various brain areas by direct projections as a neuromodulator. Additionally, OT is released into the circulation through the posterior pituitary gland, exerting endocrine effects on peripheral targets.^[^ ^1^ ^]^ OT was initially identified for its role in regulating reproductive and social behaviors. It has recently gained attention for its therapeutic potential in various brain disorders. Clinical and preclinical studies have suggested that OT administration may have beneficial effects on social cognition,^[^ ^2^ ^]^ anxiety,^[^ ^3^ ^]^ mood disorders,^[^ ^4^ ^]^ eating behaviors,^[^ ^5^ ^]^ and other neuropsychiatric disorders such as autism spectrum disorder (ASD),^[^ ^6^ ^]^ major depressive disorder, and post‐traumatic stress disorder.^[^ ^7^ ^]^ The effect of exogenous OT on modulating symptoms associated with these disorders is believed to be centrally mediated, with OT acting on its receptor in the brain.^[^ ^4^, ^8^ ^]^ Oral peptide therapeutics have low absorption owing to a breakdown in the stomach, whereas injections are not preferred for continuous daily usage, and both delivery routes result in minimal penetration to the central nervous system (CNS).^[^ ^9^ ^]^ Several clinical studies have used the non‐invasive intranasal (IN) administration approach to overcome the constraints of the blood‐brain barrier (BBB) to improve OT brain penetration.^[^ ^10^ ^]^ IN delivery of drugs and proteins with a considerable molecular weight resulted in significant brain penetration along the perivascular space of blood vessels connected to the olfactory and trigeminal nerves.^[^ ^11^ ^]^ Thus, following the IN delivery of therapeutic peptides, substantial data confirm brain penetrance via nose‐to‐brain (NTB) delivery pathways.^[^ ^12^ ^]^

Recent advances in nanotechnology have greatly improved the delivery of potential therapeutic agents using different nanocarriers. Polymeric nanocarriers of natural or synthetic biodegradable polymers have recently attracted significant research attention for delivering drugs to the brain via NTB pathways.^[^ ^12^, ^13^, ^14^, ^15^ ^]^ Poly(lactic‐co‐glycolic acid) (PLGA)based nanoparticles (NPs), composed of a biodegradable copolymer, have been extensively investigated as delivery vehicles for peptide‐based drugs. They are biocompatible and have been approved by the FDA and the European Medicines Agency.^[^ ^16^, ^17^ ^]^ PLGA‐based NPs have several attractive characteristics, such as improving drug stability, the ability to protect and control the release of therapeutic payloads, the possibility for surface modification, and tunable degradation properties depending on the polymer composition and molecular weight.^[^ ^18^, ^19^ ^]^ In addition, amphiphilic block copolymers such as PLGA–PEG (polyethylene glycol), in which hydrophobic PLGA chains are covalently linked to hydrophilic PEG, have been developed to overcome some of the limitations of PLGA alone, providing improved hydrophilicity, colloidal stability, prolonged circulation, and enhanced mucus penetration.^[^ ^20^ ^]^ The ability of NPs to function as carriers for transport across various barriers depends on both their surface composition and physicochemical features, which may affect their bioadhesive behavior, penetration properties, or endocytic absorption. It is reasonable to assume that particle size (PS) and surface charge may play a crucial role in the ability of nanocarriers to overcome mucus barriers and well‐organized epithelia. Regarding NTB delivery, smaller NPs can encounter less resistance to penetration through the mucosal layer and diffusion along the absorption path, thereby providing better opportunities for improved drug delivery to the brain after IN administration.^[^ ^21^ ^]^

The use of PLGA NPs faces a few limitations because of their low loading capacity and poor entrapment of hydrophilic drugs. It is essential to control the physicochemical properties of the developed NPs, such as PS, surface charge and drug loading (DL%) since these parameters control their biological fate, biodistribution, toxicity, and therapeutic potential.^[^ ^22^, ^23^ ^]^ Obtaining an optimized nano‐formulation using a conventional screening method (evaluating the effect of one experimental variable at a time) is expensive and time‐consuming. Therefore, the application of statistical design of experiments (DoE), such as a 3^3^ Box‐Behnken design could be a suitable approach to optimize the OT nanoformulation. DoE provides a validated and useful tool for the development and optimization of experimental procedures with fewer observations while still providing the desired information on the correlation between the experimental and response variables. The obtained model can then be used for predicting future observations within the original design range.^[^ ^24^ ^]^ This predictive and data‐driven approach parallels the principles of artificial intelligence (AI), where algorithms are trained on experimental data to learn patterns and generate reliable predictions. Among the different types of designs, a 3^3^ factorial Box Behnken design was selected in this study, as it provides a rapid and reliable means to simultaneously evaluate multiple experimental variables, efficiently capture their interactions, and optimize formulation parameters. This approach also reflects an early yet powerful form of AI‐like predictive analytics in NP research.

To evaluate the in vivo organ biodistribution of the optimized OT nanoformulation, OT was synthesized via solid‐phase peptide synthesis (SPPS) and radiolabeled with [^14^C]. SPPS is a well‐established method for preparing synthetic peptides, including OT, which offers advantages such as rapid and efficient coupling reactions by using an excess of activated amino acids that can be easily washed away. By employing the widely used fluorenyl methoxycarbonyl (Fmoc) protection strategy, SPPS facilitates the synthesis of peptides on a solid support and their subsequent cleavage, providing a reliable means of synthesizing the desired peptide chain. Carbon‐14 labelling allows sensitive detection via scintillation counting and is utilized to track the NTB delivery of OT. This strategy allows us to assess the organ biodistribution and penetration into specific brain regions of exogenous [^14^C] OT following IN administration.

The overall goal of this study is to improve brain targeting and availability of OT in the brain using an optimized OT nanoformulation via NTB delivery. Specifically, the study aims to optimize the formulation process using advanced strategies such as an AI‐based predictive approach and to characterize the physicochemical properties of OT‐loaded NPs. In addition, the in vitro and in vivo performance of OT‐loaded NPs are evaluated, including cytotoxicity, stability, release kinetics, and brain biodistribution using [^14^C]‐labeled OT NPs. By optimizing the delivery of OT to the brain via IN administration, this study aims to contribute to the development of more effective therapeutic strategies for treating ASD and other neuropsychiatric disorders.

## 2 Results

The design of polymeric nanocarriers for IN delivery of peptides to the brain tissues is one of the main concerns nowadays. This study aimed to encapsulate OT in PLGA or PLGA‐PEG NPs to achieve a suitable nanoparticulate system for IN delivery of OT to the brain tissues for central therapeutic effects. This was achieved by optimizing the formulation conditions to obtain the highest possible DL% together with a PS of ~≈100 nm to enhance NTB delivery of OT.

### 2.1 Design of Experiments for OT‐NP Formulation

**Figure 1:**
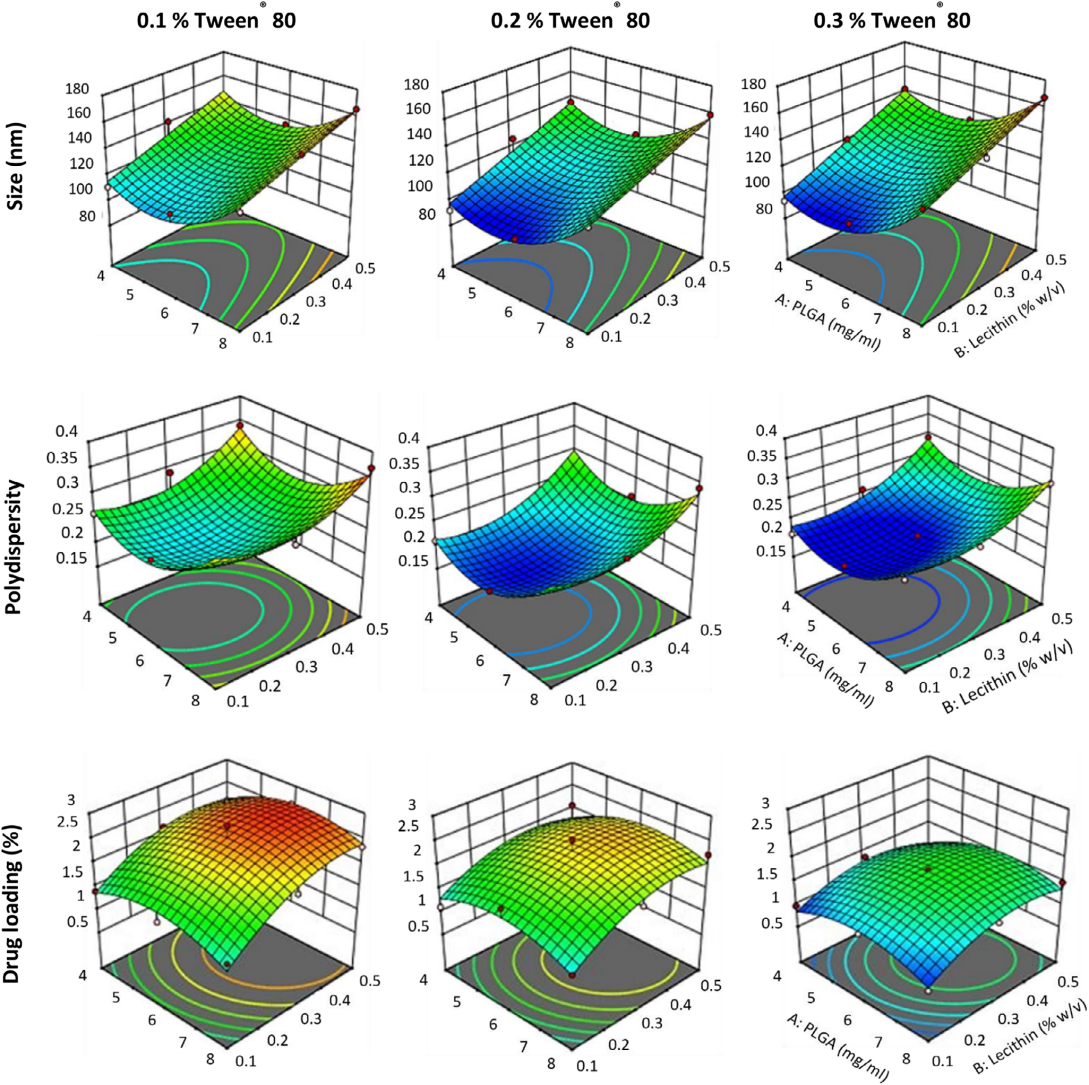
Optimization of OT‐NP design using 3^3^ factorial designs. 3D surface plots illustrating the effects of PLGA and Lecithin concentrations on size, PDI and % drug loading at three levels of Tween 80 concentration (w/v). Optimizing OT‐NP using a 3^3^ factorial design achieved the desired physicochemical characteristics: PS (116.30 nm), low PDI (0.180), and high DL% (2.76 %). Experimental values closely matched predicted values, using the optimized conditions confirming the validity of the model. The 0.2 % Tween 80 (w/v) was used for subsequent studies.

The physicochemical properties of nanocarriers significantly influence their ability to penetrate organized epithelial and mucus barriers. DoE is a mathematical approach for optimizing studies and predicting factor–factor interactions in multi‐variable investigations. Following preliminary screening and optimization of formulation parameters (Table S1, Supporting Information), a response surface methodology using a 3^3^ factorial design was employed to evaluate the effects and interactions of PLGA (X1), lecithin (X2), and Polysorbate 80 (Tween 80) (X3) concentrations on PS (Y1), polydispersity index (Y2), and drug loading (DL%, Y3). The composition of fabricated formulations and their corresponding responses are presented in Table S2 (Supporting Information). Data analysis was performed using Design‐Expert software, which generated three‐dimensional response surface and contour plots (**Figure** **1**) based on polynomial equations for each dependent variable. These equations described the relationship between process parameters and formulation characteristics, with positive and negative coefficients indicating direct and inverse effects, respectively. Statistical validity of the polynomial models was confirmed by ANOVA. All parameters fitted the quadratic model with P < 0.0001. The predicted and adjusted R^2^ values were >0.8, and the adequate precision exceeded 15, indicating a high signal‐to‐noise ratio and confirming the models’ suitability for navigating the design space.

#### 2.1.1 Effect of Formulation Parameters on PS

PS is a critical factor for NP‐based drug delivery systems. It is one of the factors which controls the kinetics of drug release, clearance profile, and internalization by target cells. The PS values from F1 to F27 showed a wide variation in response ranging between 91.52 and167.32 nm. Conclusions were drawn using polynomial equations after considering the magnitudes of coefficients and the mathematical sign they carry, i.e., positive or negative. A positive sign indicates a synergistic effect, whereas a negative sign signifies an antagonistic effect (Equation 1).

(1) $$\begin{array}{c} Y1=107.25+12.03\;X1+17.64\;X2\;-\;3.87\;X3\;-1.59\;X1X2+\\ 2.23\;X1X3+2.78\;X2\;X3+20.91\;X12+0.1500\;X22+9.01\;X32 \end{array}$$

The PS equation, 3D surface plots (Figure 1), contour plots (Figure S1, Supporting Information), and Table S3 (Supporting Information) reveal that both PLGA and lecithin concentrations have a significant (*p* < 0.0001) positive effect on PS, whereas Tween 80 concentration has a significant (*p* < 0.05) negative effect on size, and no significant interaction between the tested factors was detected. The decrease in size upon increasing Tween 80 concentration may be attributed to the prevention of PS aggregation during NP production.^[^ ^25^ ^]^ On the other side, increasing PLGA concentration results in increased viscosity, thus reducing shear stress and limiting organic solvent diffusion into the aqueous phase, which eventually results in larger particles.

#### 2.1.2 Effect of Formulation Parameters on PDI

PDI indicates PS distribution, ranging from 0 (uniform) to 1 (highly polydisperse), with values <0.3 being acceptable for polymeric NPs. Prepared formulations showed PDIs of 0.124–0.690. All parameters significantly (*p* < 0.0001) affected PDI (Table S4, Supporting Information). Effects are illustrated in 3D surface plots (Figure 1) and contour plots (Figure S2, Supporting Information).^[^ ^29^ ^]^ PLGA and lecithin increased PDI, while Tween 80 reduced it by enhancing NP stability and preventing aggregation.^[^ ^25^ ^]^

(2) $$\begin{array}{c} Y2=0.1723+0.0216\;X1+0.0365\;X2\;-\;0.0309\;X3\;-\\ 0.0070\;X1X2+0.0030\;X1X3+0.0089\;X2\;X3+\\ 0.0617\;X12+0.0428\;X22+0.0118\;X32 \end{array}$$

#### 2.1.3 Effect of Formulation Parameters on DL%

The DL% ranged from 0.76% to 2.76%, with its dependence on process parameters shown as 3D surface plots (Figure 1), contour plots (Figure S3, Supporting Information), and the polynomial equation (Equation 3). Table S5 (Supporting Information) indicates that lecithin and Tween 80 had significant positive and negative effects (*p* < 0.0001) on DL%, respectively, while PLGA concentration had no significant effect, likely due to the high drug concentration used. The negative impact of Tween 80 may result from changes in viscosity and droplet size as well as increasing OT solubility in the external phase. Whereas lecithin likely enhances DL% by acting as a molecular barrier, retaining the drug within the polymeric matrix.^[^ ^26^ ^]^

(3) $$\begin{array}{c} Y3=2.30+0.0611\;X1+0.3200\;X2\;-\;0.3994\;X3+\\ 0.0800\;X1X2+0.0158\;X1X3\;-0.1450X2\;X3\;-\\ 0.0.3600\;X12\;-\;0.3567\;X22\;-\;0.1883\;X32 \end{array}$$

#### 2.1.4 Desirability and Validation of the Model

The desirability function, a multi‐criteria optimization tool, was used to identify the best combination of operating variables for OT‐NP preparation by transforming multiple responses into a geometric mean. The optimized formulation consisted of 4 mg OT in 5 mL aqueous phase with 0.1% Tween 80 (w/v), and an organic phase containing PLGA or PLGA‐PEG (6 mg mL^−1^) with soy lecithin (0.3% w/v) in 2.5 mL acetone/ethanol (80:20 v/v), yielding a maximum desirability score of 0.8276 (Figure S4, Supporting Information), indicating strong agreement between predicted and experimental values. The regression model was statistically significant (*p* < 0.0001) with R^2^ values of 0.953 (PS), 0.940 (PDI), and 0.914 (DL%), and the optimized OT‐NP values fell within the ranges listed in Table S6 (Supporting Information). Experimental validation showed 92.6%, 95.5%, and 115.9% agreement for PS, PDI, and DL%, respectively, with a zeta potential of –32.6 mV. Following OT‐NP optimization, surface‐modified PLGA nanoparticles (OT‐NP‐PEG) were prepared under the same conditions using PEGylated PLGA to enable comparative in vitro analyses, including diffusion studies in simulated nasal media (SNM), and to evaluate organ biodistribution in mice.

### 2.2 Physicochemical Characterization of OT‐NP and OT‐NP‐PEG

**Figure 2:**
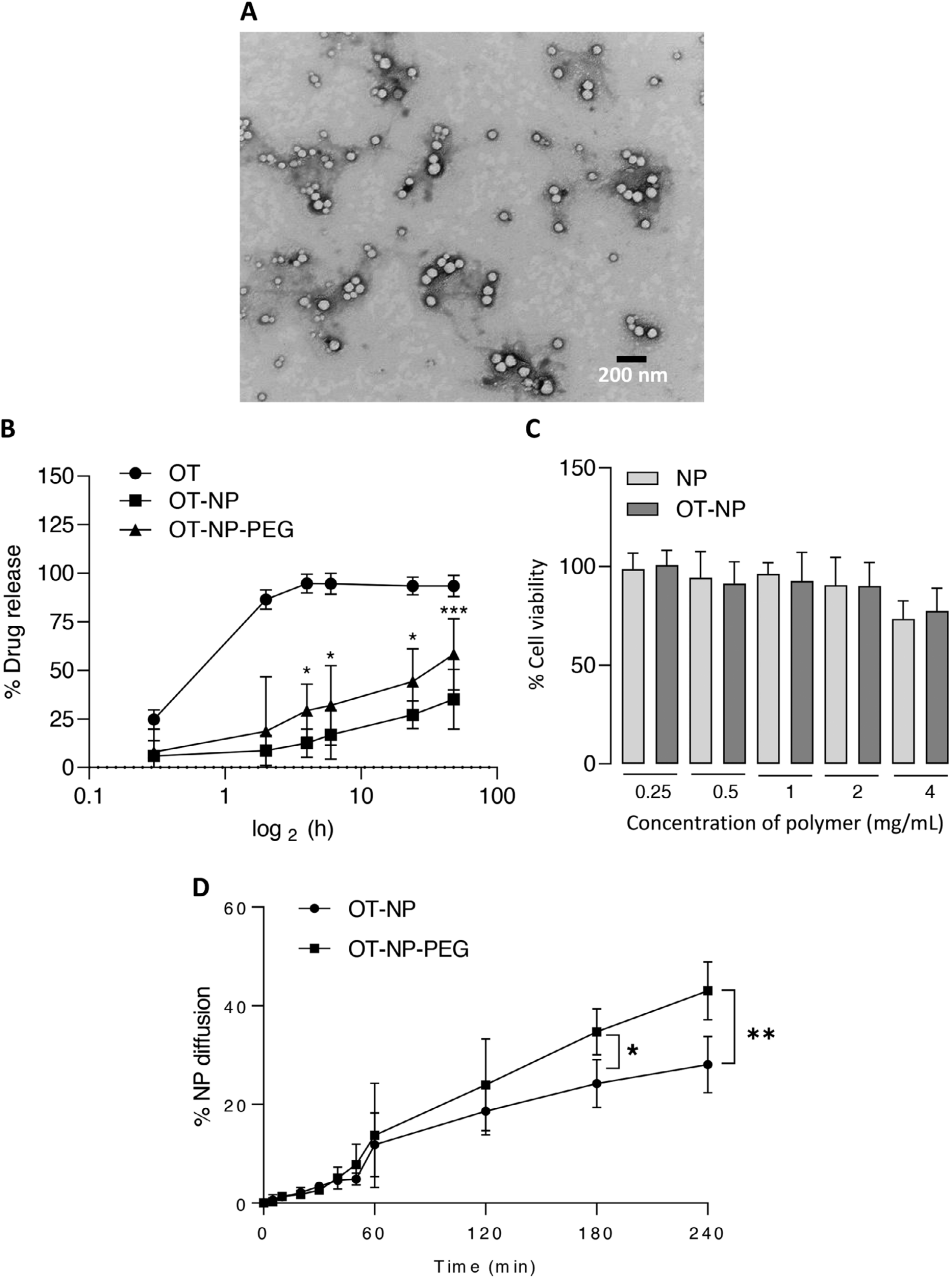
In silico and in vitro properties of OT‐NP and OT‐NP‐PEG. TEM image A) OT‐NP‐PEG shows spherical morphology and homogeneous small PS. B) Release profile of OT‐NP and OT‐NP‐PEG measured by RP‐HPLC and dialyzed against PBS at pH 7 for 48 h with stirring at 100 rpm. C) Cell viability against RPMI 2650 nasal epithelial cells after 72 h of incubation by MTT assay (*n* = 5). D) Diffusion of fluorescently labelled OT‐NP or OT‐NP‐PEG across SNM using Transwell inserts. Data from the release profile and diffusion study are expressed as mean ± SD, (n = 3). *:*p* < 0.05, **:*p* < 0.01, ***p < 0.001, Two‐way ANOVA followed by Sidak's multiple comparison test.

The prepared OT‐NP‐PEG exhibited a PS of 92.53 ± 4.5 nm, PDI of 0.21 ± 0.04, zeta potential of –21.4 ± 4.2 mV, and DL% of 3.49 ± 0.39% (**Table** **1**). The smaller size suggests enhanced colloidal stability via PEG‐mediated steric stabilization, while PEG also reduced surface charge compared to OT‐NP, consistent with PLGA‐PLA‐PEG‐coated NPs.^[^ ^27^ ^]^ TEM confirmed spherical morphology with PS around 100 nm for both formulations (**Figure** **2**A), correlating with DLS data. NTA analysis provided real‐time visualization and high‐resolution size distribution,^[^ ^28^ ^]^ giving average sizes of 100.2 ± 6.8 nm (OT‐NP) and 98.1 ± 5.6 nm (OT‐NP‐PEG), slightly smaller than DLS due to methodological differences (Figuress S5, S6, Supporting Information).^[^ ^29^ ^]^ Both techniques showed narrow, monodisperse distributions.

**Table 1:** Characterization of OT‐NP and OT‐NP‐PEG.

Formulation	OT‐NP	OT‐NP‐PEG
Size (nm)^a)^, ^e)^	116.3 ± 3.1	92.53 ± 4.5
Size (nm)^b)^, ^e)^	100.2 ± 6.8	98.1 ± 5.6
PDI^a)^	0.18 ± 0.07	0.21 ± 0.04
Zeta Potential (mV)^c)^, ^e)^	−33.2 ± 1.6	−21.4 ± 2.4
Concentration (particles/mL) by NTA	2.94^e)^ ^+013^ ± 1.30^e)^ ^+012^	3.24^e)^ ^+013^ ± 1.10^e)^ ^+012^
% EE^d)^, ^e)^	10.35 ± 1.72	12. 1 ± 1.46
DL%^d)^, ^e)^	2.76 ± 0.45	3.49 ± 0.39

^a)^ Measured by dynamic light lcattering;

^b)^ Measured by nanoparticle tracking analysis;

^c)^ Zeta potential, calculated from electrophoretic mobility measurements;

^d)^ Quantified by RP‐HPLC;

^e)^ Expressed as mean ± SD (n = 3).

Over 28 days, OT‐NP and OT‐NP‐PEG remained stable in PS, PDI, DL%, and appearance, with only minor zeta potential reductions (–33 to –26 and –19 to –16 mV, respectively) (Table S7, Supporting Information) and no aggregation or phase separation, indicating good storage stability.

Drug release from NPs can follow passive diffusion and slow matrix degradation, resulting in a biphasic drug release mechanism with an initial burst and controlled release.^[^ ^30^ ^]^ OT release from OT‐NP and OT‐NP‐PEG showed a biphasic pattern. Free OT reached 100% release within 4 h, while OT‐NP released 27% and 35%, and OT‐NP‐PEG released 42% and 58% at 24 and 48 h respectively (Figure 2B), reflecting sustained release, with faster release from PEGylated NPs due to surface adsorption and better water interaction.

Diffusion through SNM was evaluated using Transwell inserts (Figure 2D). Less than 10% of OT‐NP and OT‐NP‐PEG diffused in 60 min, increasing to 26.45% and 44.2% at 4 h, respectively. OT‐NP‐PEG consistently showed higher diffusion, likely due to PEG coating that could minimize any adhesive interactions with the mucus mesh constituents.^[^ ^31^, ^32^ ^]^

### 2.3 In Vitro Cytotoxicity Study

The safety of OT‐NP on the human nasal cell line, RPMI 2650, as an in vitro cytotoxicity model was investigated using the MTT assay. The cytotoxicity assessment was performed at 72 h using an optimized cell seeding density of 2 × 10^4^ k cells per well (data not shown). NP concentrations with or without OT up to 4 mg mL^−1^ polymer or 96 µg OT mg^−1^ showed no significant reduction in cell viability after 72 h exposure (Figure 2C). Cell viability remained above 85% at all concentrations tested, indicating OT‐NP were non‐toxic to cells under the experimental conditions.

### 2.4 Characterization of Synthesized OT and [^14^C] OT

**Figure 3:**
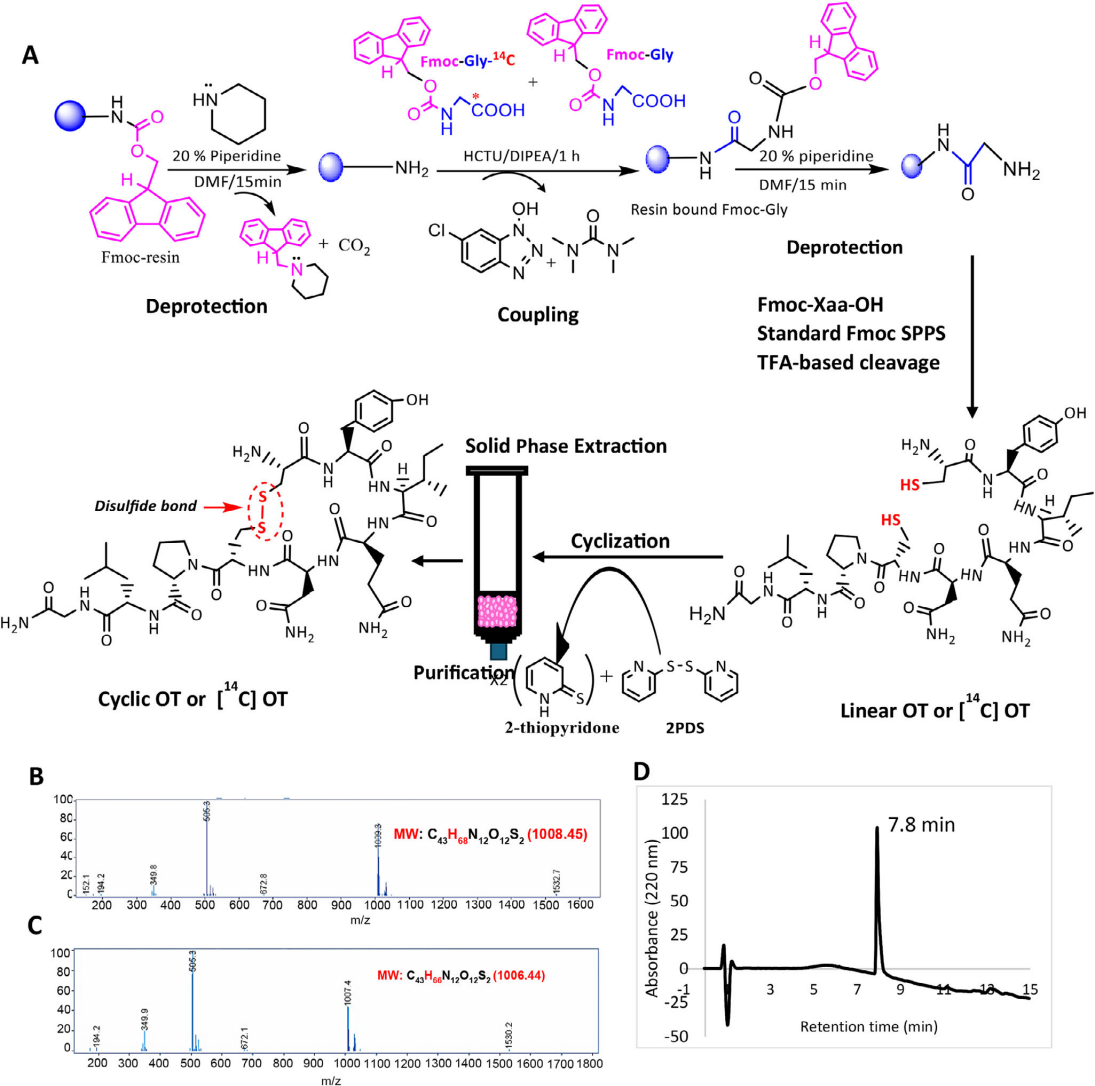
Synthesis of OT and [^14^C] OT by Fmoc SPPS. OT was synthesized on a TentaGel resin solid support A). Standard Fmoc solid‐phase peptide synthesis cycle with Fmoc‐protected amino acid residues as the building blocks, HCTU as the coupling reagent, N, N'‐Diisopropyl carbodiimide as an amino acid activator, and piperidine for Fmoc removal as a standard reagents were used. A linear OT was obtained by cleaving the peptide chains from the resin and removing all side chain‐protecting groups with trifluoroacetic acid containing the scavengers. Deprotection and coupling were onfirmed with the TNBS test. Cyclization forming disulfide bonds was carried out using 2,2′‐Bispyridyl disulfide and purified by SPE column. For [^14^C] OT synthesis, Fmoc‐glycine was replaced by Fmoc‐Gly‐ [^14^C] as mentioned in the red dotted box. LC‐MS spectrum of OT m/z calculated for (B) linear OT: 1008.45 and (C) cyclic OT: 1006.44 [M+H]^+^. Panel D shows the representative RP‐HPLC chromatogram of purified [^14^C] OT, at a detection wavelength of 220 nm. The successful synthesis of OT and [^14^C] OT was confirmed by RP‐HPLC, LC‐MS and LSC.

The characterization of the linear OT by LC‐MS resulted in a molar mass of 1008.45 (C_43_, H_68_, N_12_, O_12_, S_2_) (**Figure** **3**B), which is consistent with the molar mass of linear OT (oxidized form) (H_68_ vs H_66_) (Figure 3C). The molar mass of functional cyclic OT is 1006.44 (C_43_, H_66_, N_12_, O_12_, S_2_) due to the intramolecular S‐S bond between two cysteine amino acids forming an oxidized state (loss of two hydrogen atoms) with cyclic OT structure. After the cyclization of the linear OT using 2‐PDS, the peptide was again subjected to characterization by LC‐MS, which resulted in a molar mass of 1007.4, matching the molar mass of the commercial OT. These results confirm the successful cyclization of linear OT into a cyclic OT molecule.

The purification was confirmed by analyzing the purified OT sample using RP‐HPLC, which resulted in a clean OT peak with a similar retention time (7 min) to that of commercial OT. The yield of the dried OT by mass was 94% and 87% for small and large synthesis scales, respectively. The synthesized OT sample was analyzed by RP‐HPLC, and the final purified yield was calculated as 84% and 90.67% for 0.05 and 0.0125 mmole g^−1^ (Table S8, Supporting Information) synthesis scales, respectively. The purified [^14^C] OT was analyzed by RP‐HPLC, which resulted in a final yield of 74.4% (Table S9, Supporting Information), while the yield of [^14^C] OT by mass was 94.6%. Representative RP‐HPLC chromatograms of purified [^14^C] OT, characterized by RP‐HPLC are shown in Figure 3D. The radiochemical yield was calculated as 53.35% from the standard calibration curve using RP‐HPLC and LSC. A specific RA of 4.45 µCi mg^−1^ was obtained, sufficient for the required dosing in pharmacokinetic profiling in mice.

### 2.5 Biodistribution of [^14^C] OT and [^14^C] OT‐NP‐PEG in Nasal Tissues and Brain Post‐IN Administration

**Figure 4:**
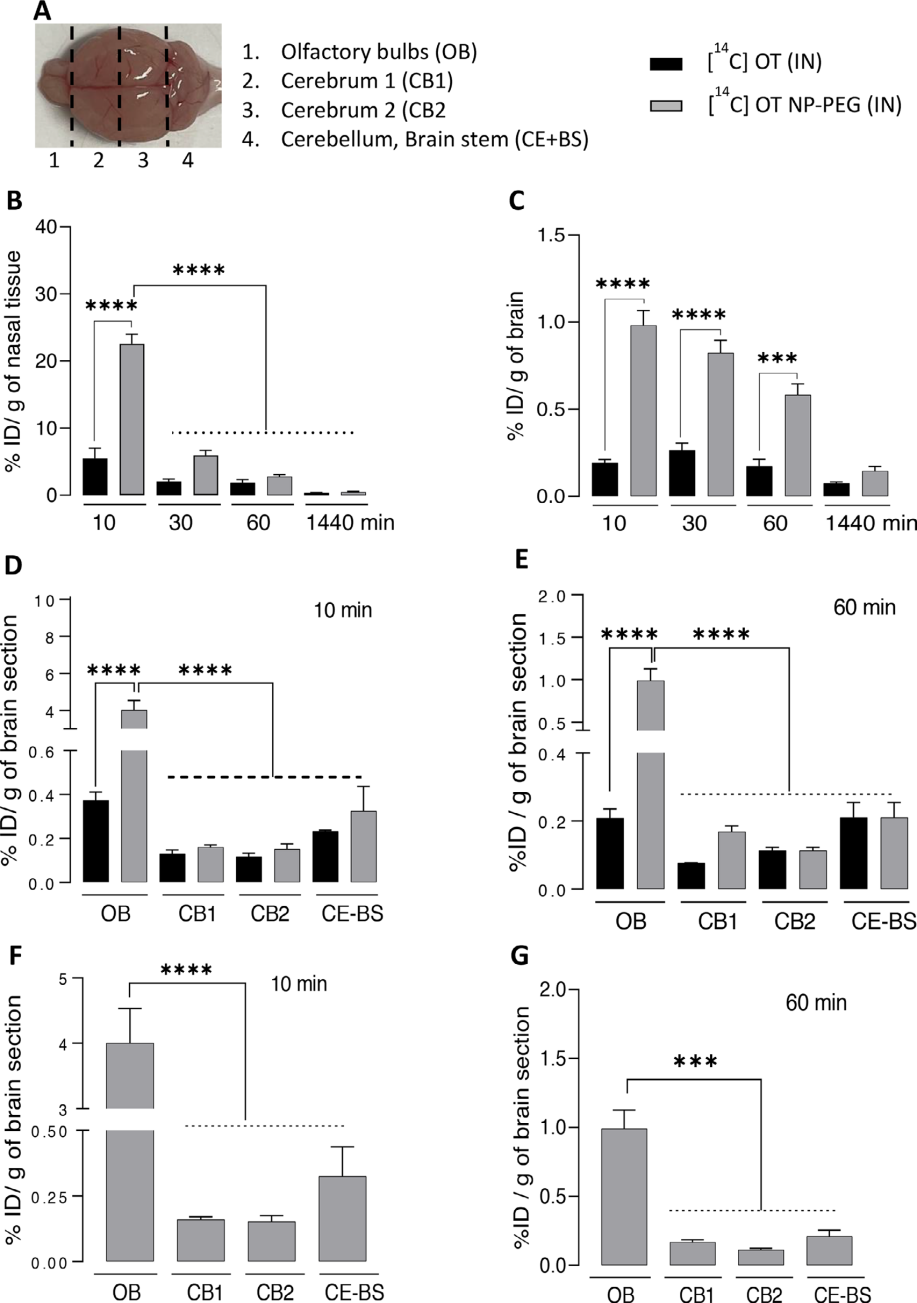
Brain uptake of [^14^C] OT and [^14^C] OT‐NP‐PEG after IN administration. Mice were administered 20 µl (10 µl each nostril) of [^14^ C] OT or [^14^ C] OT‐NP‐PEG in PBS under inhalation anesthesia. At the terminal time point (10 or 60 min), mice were euthanized under terminal anesthesia, and blood was withdrawn from the inferior vena cava. The different brain segments A) were dissected and processed for LSC. The % IDg^−1^ tissues of nasal tissues B), brain C) is presented for soluble [^14^C] OT or [^14^C] OT‐NP‐PEG. The % IDg^−1^ brain segment of [^14^ C] OT or [^14^C] OT‐NP‐PEG (10 min) (D) or [^14^C] OT or [^14^C] OT‐NP‐PEG (60 min) E), [^14^C] OT‐NP‐PEG (10 min) F) or [^14^C] OT‐NP‐PEG (60 min) G) is presented. Data are expressed as mean ± SEM, n = 3. ***p < 0.001; ****p < 0.0001 (One‐way ANOVA followed by Tukey's multiple comparison test).

The biodistribution profiles of [^14^C] OT or [^14^C] OT‐NP‐PEG as percentage injected dose per gram (% IDg^−1^) of brain and nasal cavity samples are shown in **Figure** **4**. IN administration of [^14^C] OT‐NP‐PEG resulted in more effective brain uptake than soluble [^14^C] OT, with corresponding %ID values of 0.98 ± 0.08 (****p < 0.0001), 0.82 ± 0.07 (****p < 0.0001), and 0.58 ± 0.06 (***p < 0.001), compared with 0.19 ± 0.01, 0.26 ± 0.04, and 0.17 ± 0.04 % IDg^−1^ at 10, 30, and 60 min, respectively (Figure 4C). This could be due to higher retention in the nasal cavity of [^14^C] OT‐NP‐PEG compared to soluble [^14^C] OT at all time points tested (Figure 4B). These findings indicate the possibility of potential benefits of the NP formulation possessing appropriate physicochemical characteristics for enhanced [^14^C] OT‐NP‐PEG uptake in the brain.

### 2.6 Biodistribution of [^14^C] OT and [^14^C] OT‐NP‐PEG in Peripheral Organs

Figure S8 (Supporting Information) illustrates the overall in vivo whole‐organ biodistribution of [^14^C] OT (Figure S8A, Supporting Information) and [^14^ C] OT‐NP‐PEG (Figure S8B, Supporting Information) after IN administration. [^14^C] OT‐NP‐PEG exhibited higher lung uptake than [^14^C] OT initially, but both decreased over time (Figure S9A, Supporting Information). In the liver, [^14^C] OT showed significantly greater accumulation than [^14^C] OT‐NP‐PEG at 10, 30 and 60 min, indicating higher systemic exposure (Figure S9B, Supporting Information), while both formulations had low and comparable uptake in the spleen (Figure S9C, Supporting Information). [^14^C] OT‐NP‐PEG showed higher accumulation in the heart at 10 min compared to [^14^C] OT, with these differences disappearing over time (Figure S10A, Supporting Information). The stomach showed comparable uptake for both [^14^C] OT and [^14^C] OT‐NP‐PEG, with a noticeable drop after 30 min (Figure S10B, Supporting Information). In the intestine, [^14^C] OT exhibited significantly higher uptake compared to [^14^C] OT‐NP‐PEG at 30 min (Figure S10C, Supporting Information). Collectively these findings suggest that [^14^ C] OT‐NP‐PEG differs in its peripheral biodistribution profile, with [^14^ C] OT generally exhibiting higher accumulation than ^14^[C] OT‐NP‐PEG in liver, indicating reduced systemic exposure compared to [^14^ C] OT.

### 2.7 Enhanced Nasal and Brain Uptake of OT‐NP‐PEG Following IN versus IV Administration

A comparative analysis revealed that IN administration achieved significantly higher OT‐NP‐PEG uptake in nasal and brain tissues than IV administration (Figure S11, Supporting Information). In nasal tissues, % IDg^−1^ values after IN administration were 22.56 ± 1.44% at 10 min (****p < 0.0001) and 5.97 ± 0.71% at 30 min (***p < 0.001), far exceeding those observed after IV administration (Figure S11A, Supporting Information). Similarly, brain uptake was significantly greater with IN administration, reaching 0.98 ± 0.08% at 10 min (****p < 0.0001), 0.82 ± 0.07% at 30 min (****p < 0.0001), and 0.58 ± 0.06% at 60 min (***p < 0.001) (Figure S11B, Supporting Information). These findings highlight the potential of the IN route as a non‐invasive strategy for brain‐targeted delivery.

### 2.8 Spatial Brain Distribution of [^14^C] OT and [^14^C] OT‐NP‐PEG after IN Administration in Mice

The transport of [^14^C] OT‐NP‐PEG in different brain regions after IN administration was investigated. Radioactivity in the brain segments, the olfactory bulbs (OB), the cerebrum (CB 1 and 2), the brain stem (BS), and the cerebellum (CE) (Figure 4A) were determined at 10 and 60 min after IN administration. Figure 4D illustrates that the highest uptake was detected in the OB of mice treated with IN [^14^C] OT‐NP‐PEG at 10 min, suggesting a rapid uptake from the nasal cavity into the brain. [^14^C] OT‐NP‐PEG achieved brain values of 4.00 ± 0.52% IDg^−1^ compared to only 0.37 ± 0.03% IDg^−1^ for soluble [^14^ C] OT at 10 min (Figure 4E). [^14^C] OT‐NP‐PEG uptake in the OB section was significantly higher than in other brain sections at both 10 and 60 min (Figure 4F,G). This finding suggests a direct brain uptake of [^14^C] OT‐NP‐PEG via olfactory nerve pathway. OT could still be observed in the olfactory brain region for up to 60 min, suggesting a continuous uptake from the nasal cavity. These findings highlight that IN administration of [^14^C] OT‐NP‐PEG can successfully target the brain, presenting a potential delivery strategy for treating neurological and neuropsychiatric disorders.

### 2.9 Effect of OT and OT‐NP‐PEG on Self‐Grooming Behaviors in Mice After IN Administration

**Figure 5:**
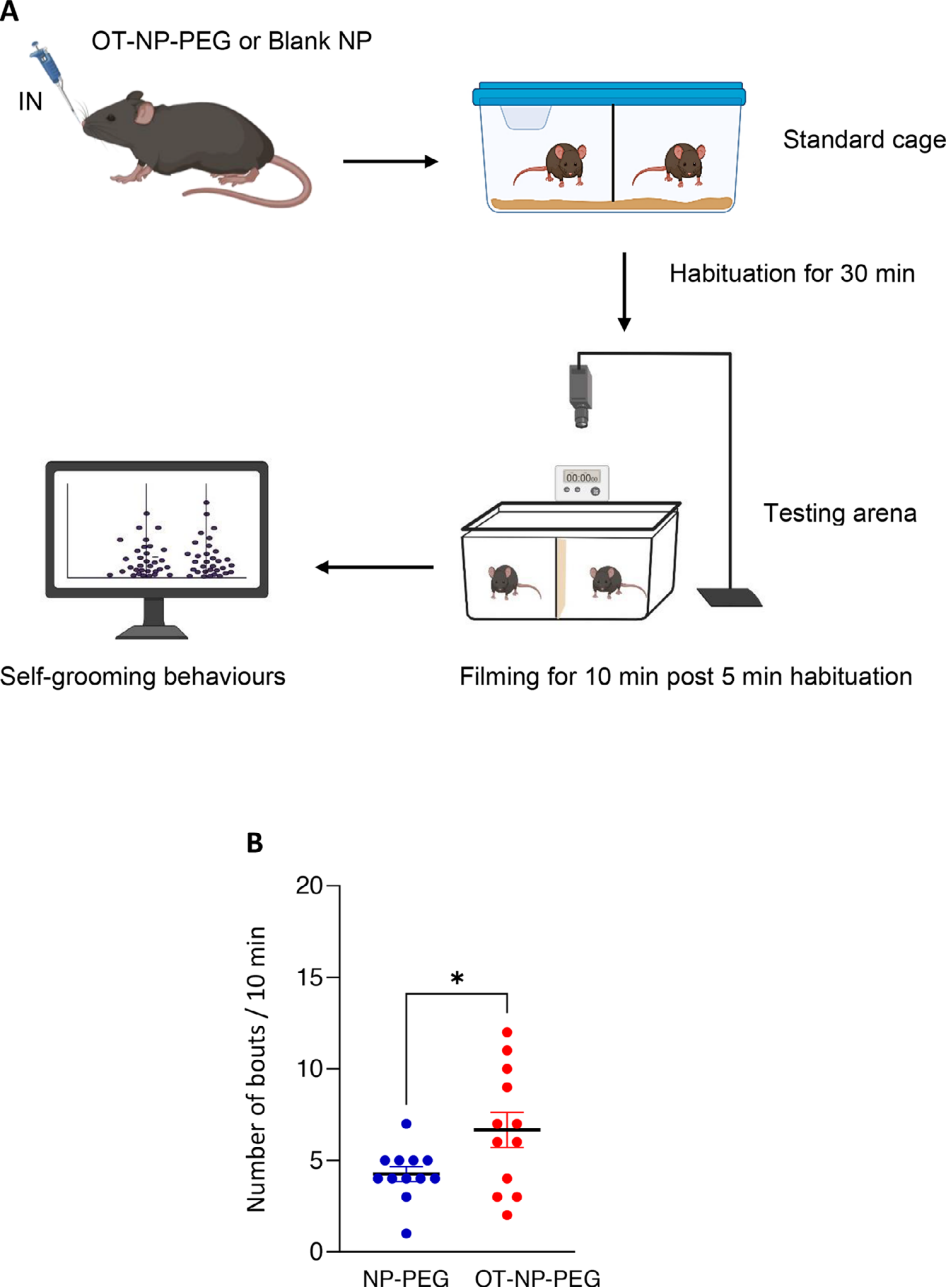
Effect of OT‐NP‐PEG and blank NP on self‐grooming behaviours in mice after IN administration. Schematic representation of the self‐grooming behavioral experiment A). Frequency of self‐grooming behaviour (number of bouts) of IN blank NP (5 µL volume in sterile PBS) or OT‐NP‐PEG (equivalent to 0.7 mg kg^−1^ OT in 5 µL sterile PBS) B), scored for 10 min. The dosing and analyses were carried out in a blinded manner. A higher level of self‐grooming frequency by IN OT‐NP‐PEG was observed compared to the blank NP. Data are expressed as mean ± SEM, n = 12, *p < 0.05; (Unpaired Student's *t*‐test).

The results demonstrate that OT‐NP‐PEG (0.7 mg kg^−1^ OT) significantly increased the frequency of self‐grooming bouts within a 10 min observation period compared to blank NPs after IN administration (**Figure** **5**). This behavioral effect is further supported by our spatiotemporal uptake study (Figure 4), which demonstrated that OT‐NP‐PEG reached the OB within 10 min of IN administration and remained detectable in other brain regions up to 60 min. These findings show that the improved brain delivery of OT‐NP‐PEG underlies the robust increase in self‐grooming behaviors (number of bouts), highlighting the effectiveness of this delivery strategy for targeting the brain.

## 3 Discussion

IN OT administration has been explored in clinical trials for various neuropsychiatric disorders for which we currently lack effective treatments, including ASD, schizophrenia, and Prader‐Willi syndrome, all of which are characterized by impaired social functioning.^[^ ^33^, ^34^ ^]^ However, many trials have yielded inconsistent results,^[^ ^35^, ^36^, ^37^ ^]^ while the overall volume of evidence suggests that IN OT has a positive effect.^[^ ^38^ ^]^ This contrasts with the consistent beneficial effects observed in animal models,^[^ ^39^, ^40^ ^]^ where OT is often administered directly into the brain.^[^ ^41^, ^42^ ^]^ The inconsistency in human studies may result from the poor bioavailability of OT delivered to the brain with standard IN sprays and highlights the need for improved delivery methods to enhance the therapeutic potential of OT. NPs based on FDA‐approved polymers such as PLGA and PLGA‐PEG have gained attention due to their biocompatibility, stability, and controlled release capabilities.^[^ ^43^ ^]^ These properties make them promising candidates for enhancing OT delivery to the brain, potentially overcoming the challenges associated with peripheral side effects and improving brain targeting efficiency. These challenges emphasize the need for advanced delivery platforms capable of improving OT bioavailability in the brain. In this context, FDA‐approved PLGA‐based polymers have emerged as promising candidates due to their biocompatibility, stability, and ability to sustain peptide release.

Encapsulating OT in nanoparticulate systems has been studied to improve its bioavailability and brain targeting via IN administration. Encapsulation within polymeric NPs provides a stable environment that is crucial for maintaining the potency of peptide drugs.^[^ ^44^ ^]^ Free OT is a small, hydrophilic peptide that undergoes rapid proteolytic degradation in nasal and systemic tissues, resulting in a plasma half‐life of only 3‐6 minutes.^[^ ^45^ ^]^ Encapsulation within PLGA protects OT from enzymatic degradation and enables sustained release of OT.^[^ ^46^ ^]^ In addition, the nanoscale size of the PLGA‐based NP formulation facilitates unhindered diffusion through the nasal mucus mesh and across the olfactory epithelium, reducing enzymatic clearance and enhancing access to NTB transport pathways. Nanoparticles within the 50–200 nm range can penetrate through the mucus mesh without becoming sterically trapped, whereas larger particles (>200 nm) are hindered by mucin fibers and cleared more rapidly ^[^ ^32^ ^]^ It is reasonable to assume that smaller particles encounter less resistance to mucosal penetration and absorption pathway diffusion, thereby improving the probability of enhanced drug delivery to the brain following IN administration.^[^ ^21^ ^]^

However, many existing OT nanoparticle studies face limitations, particularly in terms of insufficient brain biodistribution data and suboptimal pharmacokinetic profiles. For example, previous research using bovine serum albumin (BSA)‐based NPs conjugated with transferrin (Tf‐OT‐NP) reported positive effects on seizure severity and hippocampal neurogenesis in pentylenetetrazol‐induced epileptic rats but did not provide a detailed pharmacokinetic analysis or biodistribution data, leaving uncertainties about the actual uptake of OT‐loaded NPs in the brain.^[^ ^47^ ^]^ Similarly, OT‐loaded NPs using PLGA and BSA conjugated with transferrin (Tf) or rabies virus glycoprotein (RVG) demonstrated favorable release profiles and enhanced BBB transport in an in vitro model.^[^ ^48^ ^]^ OT‐loaded chitosan nanoparticles (O‐CSNP) have been previously formulated and characterized. The O‐CSNP displayed nano‐spherical particles (30–50 nm) and improved release profiles, but the study lacked a detailed in vivo biodistribution analysis.^[^ ^49^ ^]^ Another study encapsulated OT using double and simple emulsion methods, yielding NP sizes of 195 and 226 nm, and OT loadings of 4% and 3.3%, respectively.^[^ ^50^ ^]^ A study utilized a non‐aqueous nanoprecipitation method that encapsulated the OT in poly (sebacic anhydride) NPs, resulting in spherical particles ≈300 nm in size. The OT‐loaded NPs exhibited a burst release of ≈50% within the first hour in an aqueous medium, suggesting their potential for targeted delivery to the olfactory epithelium using a nasal atomizer.^[^ ^51^ ^]^

The NPs’ size and surface charge are critical factors influencing their ability to reach the CNS, especially via NTB delivery pathway. The physiological characteristics of this route favor smaller NPs, as they can navigate the nasal mucosa and reach the brain more efficiently.^[^ ^52^ ^]^ Research has demonstrated that smaller NPs result in greater brain accumulation and superior therapeutic outcomes compared to larger ones. For example, in a study involving an epilepsy rat model, 100 nm PEG‐PLA NPs administered intranasally led to higher brain accumulation and improved therapeutic efficacy compared to 500 nm NPs.^[^ ^53^ ^]^ In addition to PS, the presence of PEG on the surface of NPs may have contributed to the enhanced NTB delivery. The surfactant‐like properties of PEG, as well as its interaction with endothelial cells, have been suggested to increase NP diffusion through biological barriers such as the olfactory mucosa.^[^ ^54^ ^]^ Furthermore, PEG's hydrophilicity reduces interactions with nasal mucus, facilitating improved diffusion across the nasal epithelium and into the brain.^[^ ^55^ ^]^

None of the above studies provided evidence of the NP's distribution in specific brain regions or of the peripheral organ distribution after IN administration. It is essential to assess the amount of OT that reaches the brain, particularly following IN administration, as this non‐invasive route can exhibit variable efficacy depending on the dose and delivery method. Without a clear understanding of how much OT reaches the target regions, predicting or controlling therapeutic effects becomes challenging. Therefore, accurate monitoring of OT levels in the brain is critical to ensure optimal therapeutic outcomes and to adjust dosing strategies accordingly.

Our OT‐NP‐PEG formulation exhibited a negative surface charge (−21 to −33 mV), which likely facilitated its preferential movement via the olfactory route.^[^ ^56^ ^]^ This behavior arises from the abundance of negatively charged sialic‐acid residues in nasal mucus and the olfactory epithelium and consequently, anionic nanoparticles undergo electrostatic repulsion, leading to reduced mucoadhesion and limited binding to mucins.^[^ ^56^ ^]^ Such reduced interaction increases the likelihood of diffusion toward the olfactory region and supports subsequent engagement of olfactory nerve–associated transport mechanisms. Particle size further influences the ability of nanoparticles to penetrate nasal mucus. PEGylation also increases hydrophilicity and minimies nonspecific interactions with mucin within the mucosal environment.^[^ ^57^ ^]^ PEGylated nanoparticles of ≈100 nm can penetrate nasal mucus via Brownian motion because their size is significantly smaller than the typical mucus pore spacing (estimated to be 100–500 nm) Furthermore, our DoE optimization was based on formulation parameters that directly influence the physicochemical characteristics required for effective NTB delivery. The polymer concentration, lecithin content, and Tween 80 levels were systematically varied because these factors determine particle size, uniformity, and drug loading. These factors influence nanoparticle stability, dispersion behavior, and subsequent mucosal transport. Rather than imposing a predetermined target size, a desirability function was applied to balance reducing particle size and PDI with maximizing drug loading. Through this data‐driven process, the DoE identified a formulation producing ≈100 nm, narrowly dispersed nanoparticles (low PDI) and a maximum DL of 3.4%. The resulting formulation showed improved diffusion in simulated nasal mucus and significantly higher brain uptake in vivo. These results are consistent with previous studies demonstrating the value of structured optimization approaches in guiding nanoparticle design for mucosal and brain delivery.^[^ ^32^, ^57^ ^]^

Another advantage of our study was the application of [^14^C] radiolabeling to precisely track OT‐NP‐PEG throughout the brain and peripheral organs, demonstrating significantly enhanced brain uptake and retention, particularly in the olfactory bulb and frontal cortex. This is a major advancement, as most earlier studies focused primarily on CSF or plasma OT levels without offering insights into the specific regions of the brain affected by OT administration. The use of [^14^C] for radiolabeling peptides, including OT, is well‐documented, although its application to OT encapsulated in NPs for NTB delivery has not been extensively reported. The [^14^C] Labelling offers advantages such as low energy emission, minimal tissue damage, and cost‐effectiveness.^[^ ^58^ ^]^ Although, alternative radioisotopes such as tritium (^3^H) and iodine‐125 can be used for peptide labelling, the latter typically requires the incorporation of iodine‐125 into tyrosine or histidine residues in the peptide or protein while the former suffers from higher quenching in tissues.^[^ ^59^, ^60^ ^]^ In contrast, [^14^C] labelling provides a distinct advantage due to its direct positioning within the drug's core, thereby reducing the risk of drug loss or the need for modification.

In addition to these established benefits, our choice of using ^14^C instead of ^3^H labelling was based on its enhanced chemical stability and reliability for quantitative pharmacokinetic measurements. The covalent C─C bond in the ^14^C isotope maintains its stability during metabolic processes, thus inhibiting label removal or isotope exchange. However, 3H labelling is more prone to partial hydrogen exchange with solvent or adjacent biomolecules, yielding inaccurate biodistribution data and quenching‐related errors in high‐protein tissues, including the brain.^[^ ^1^, ^2^ ^]^ Additionally, the low β‐energy emission of ^14^C (E_max_ = 0.156 MeV) facilitates highly effective liquid scintillation counting with minimal self‐absorption, leading to superior signal linearity and lower background noise in comparison to high‐energy isotopes.^[^ ^1^ ^]^ More importantly, the generation of scintillation photons per decay for ^14^C is approximately eightfold more than ^3^H (E_max_ = 0.019 MeV), which results in improved assay sensitivity and enhanced counting efficiency in tissue samples.^[^ ^3^ ^]^ Overall, the detection benefits and physicochemical advantages establish ^14^C labelling as the preferred method for precise quantification of peptide biodistribution in vivo.^[^ ^61^ ^]^ This study represents a novel application of [^14^C]‐labelled OT in assessing the pharmacokinetics and biodistribution of [^14^ C] OT‐NP‐PEG following IN administration. Previous studies have employed various isotopes for tracking OT, primarily in rodent models. For instance, Lee et al. utilized deuterated OT (d5‐OT) to investigate its pharmacokinetics in rhesus macaques, observing increased plasma and CSF concentrations post‐IN and IV administration.^[^ ^62^ ^]^ Similar studies reported minute fractions (0.002%) of administered OT reaching the CSF, highlighting the challenges in achieving effective brain delivery.^[^ ^45^, ^63^ ^]^ The use of [^14^C] OT‐NP‐PEG in our study enabled precise quantification of OT uptake in specific brain regions, providing a novel and more accurate method for tracking brain distribution compared to conventional techniques. This approach not only demonstrated the enhanced brain targeting of OT via the NTB pathway but also addressed the critical gap in previous studies, which lacked detailed brain tissue biodistribution data. The [^14^C] labelling method thus represents a significant advancement in understanding the pharmacokinetics of OT delivery to the CNS.

The enhanced delivery and detection of OT via the NTB pathway is a critical consideration. Our study findings indicate that the amount of OT delivered via [^14^ C] OT‐NP‐PEG is successfully delivered and detected in brain tissues. This enhanced delivery is crucial for achieving the desired central therapeutic effects, particularly in treating neuropsychiatric disorders where effective brain targeting is essential. Our biodistribution study demonstrated that IN administration of [^14^C] OT‐NP‐PEG resulted in 0.98 ± 0.08, 0.82 ± 0.07, and 0.58 ± 0.06 %ID g^−1^ in the brain at 10, 30, and 60 min, respectively. For a typical mouse brain mass of ≈0.45 g, these values correspond to ≈49 , 41, and 29 ng of OT per whole brain. These amounts are several orders of magnitude higher than physiological endogenous OT levels, which are typically in the low‐picogram range (1–10 pg mL^−1^) in rodent cerebrospinal fluid or brain extracellular fluid.^[^ ^64^ ^]^ It is also important to note that quantifying endogenous OT is most relevant in OT‐deficient or knockout models, where the aim is to restore physiological concentrations. In contrast, our experiments were performed in wild‐type mice, which have an intact OT system. The purpose of our study was therefore not to normalize a deficiency, but to determine whether nanoparticle encapsulation can enhance central delivery relative to free OT. Accordingly, the negligible brain penetration observed for free [^14^C] OT (<0.1 %ID g^−1^) confirms that the elevated levels detected with [^14^C] OT‐NP‐PEG arise from improved delivery. The spatiotemporal distribution analysis of OT‐NP‐PEG in the brain also revealed rapid uptake, with the olfactory bulb exhibiting the highest levels within 10 min post‐IN administration. This aligns with previous findings where polymeric NPs encapsulating leucine‐enkephalin hydrochloride were detected in the olfactory bulb as early as 5 min post‐IN administration, further supporting the notion that the olfactory route serves as the primary entry point for NP‐based delivery systems.^[^ ^65^ ^]^ The high concentration in the OB suggests that the olfactory route is a primary pathway for nanoparticle‐based delivery systems to access the brain.

Comparative analysis with IV administration of [^14^C] OT‐NP‐PEG demonstrated significantly higher brain uptake and prolonged residence time following IN administration. This supports the advantages of the NTB delivery approach over systemic routes, providing a more targeted and efficient method for OT delivery to the brain. These findings are consistent with previous studies showing enhanced brain targeting of PLGA NPs following IN administration compared to IV administration of lamotrigine‐loaded PLGA NPs.^[^ ^66^ ^]^ Similarly, thyrotropin‐releasing hormone‐loaded PLGA NPs administered intranasally showed promising results in reducing seizure frequency and severity in a rat model of epilepsy.^[^ ^53^ ^]^ OT undergoes rapid metabolic degradation within brain tissues due to its short half‐life. Following the release of [^14^C] OT from [^14^C] OT‐NP‐PEG, it may undergo conversion to its metabolites, which still retain the carbon‐14 label. The detection of only carbon‐14 may not provide a direct correlation with the parent OT in brain tissues as the LSC only detects carbon‐14. Therefore, future pharmacokinetic studies using LC‐MS are recommended to accurately detect both the parent OT and its metabolites in brain tissues following IN administration of [^14^C] OT‐NP‐PEG.

Self‐grooming is a well‐characterized behavioral endpoint in rodents and has consistently been linked to central OT signaling. Exogenous OT, when administered centrally, has been shown to induce grooming bouts, while genetic or pharmacological disruption of OT receptor function diminishes such responses.^[^ ^67^, ^68^, ^69^ ^]^ The spatiotemporal analysis further supports this interpretation by showing rapid and preferential uptake of OT‐NP‐PEG in the OB within 10 min post IN administration. A previous study has similarly reported that IN neuropeptide‐loaded NPs accumulate in the OB within minutes, a region that provides a direct portal for further distribution to forebrain areas implicated in grooming control.^[^ ^65^ ^]^

In the preliminary behavioral study, we found that IN administration of OT‐NP‐PEG at a dose of 0.7 mg kg^−1^ (OT), significantly increased the frequency of self‐grooming behavior in mice, compared to the blank NP, which suggests central activation of OT receptors. We chose 0.7 mg kg^−1^ because it lies within the commonly used 0.3–1.0 mg kg^−1^ range that has been shown to produce central and behavioral effects in mice following systemic or intranasal administration. Given the very short plasma half‐life of OT (≈2–5 min in rodents) and its rapid enzymatic degradation, using a somewhat higher dose within standard practice is reasonable to offset rapid clearance and to improve CNS exposure. This is also supported mechanistically by RAGE‐mediated transport of circulating OT across the BBB, implying that greater plasma exposure increases brain entry.^[^ ^3^ ^]^ Additionally, because OT receptor affinity is in the low‐nanomolar range, higher brain OT levels improve the likelihood of achieving meaningful receptor occupancy in target circuits that mediate efficacy. Our dose therefore balances feasibility, prior precedent, and pharmacokinetic constraints, aiming to achieve therapeutically relevant brain concentrations without exceeding doses commonly reported in mice. Furthermore, we included blank NP (identical composition without OT) as a behavioral control to exclude any anesthesia or formulation related effects, including local sensory stimulation in the nasal cavity that could influence the frequency of self‐grooming (number of bouts).This ensured that any observed behavioral changes were not attributable to the nanoparticle carrier itself but specifically to the presence of encapsulated OT. Moreover, this study used wild‐type mice with normal endogenous OT level in the brain, our behavioral experiment served as a proof of concept to confirm that the optimized nanoparticle‐mediated delivery can elevate central OT above the physiological baseline, rather than to compare treatment efficacies. For this purpose, the radiolabeled organ biodistribution data provide the appropriate and rigorous comparison, demonstrating that only the nanoparticle formulation delivers quantifiable OT to the brain for inducing behavioral effects. The robust increase in grooming, together with the early distribution profile, provides behavioral evidence that intranasally delivered OT‐NP‐PEG efficiently bypasses the BBB to rapidly engage central OT receptors via NTB delivery pathways. Future behavioral investigations in relevant disease models will be required to evaluate the therapeutic efficacy and optimal dosing parameters in more detail.

The in vivo performance of OT‐NP‐PEG can be directly attributed to the physicochemical characteristics identified through our DoE optimization. The optimized NPs exhibited a size of ≈100 nm with a narrow size distribution, a profile known to support predictable mobility and consistent interaction with the nasal epithelium in IN delivery models.^[^ ^70^ ^]^ The PEG‐presenting surface minimized adhesive interactions within the mucus layer, enabling penetration toward the olfactory brain region.^[^ ^71^ ^]^ At the same time, the PLGA core protected OT from enzymatic degradation in the nasal cavity and provided a sustained release, a well‐established advantage of PLGA systems for peptide stabilization.^[^ ^72^ ^]^ These combined design characteristics translated into significantly enhanced distribution of OT within the olfactory bulbs and whole brain compared with free OT, demonstrating that the optimized OT nanoparticle characteristics act synergistically to improve mucosal transport, preserve peptide integrity, and facilitate efficient NTB delivery. The observed enhanced delivery and behavioral effect therefore closely reflect the formulation's optimized characteristics, supporting the rationale underlying the DoE‐driven design.

## 4 Conclusion

This study develops a nanoparticulate system for nose‐to‐brain delivery of OT to treat neuropsychiatric disorders. Using FDA‐approved PLGA polymers, nanoparticles with favorable physicochemical characteristics, biocompatibility, sustained release, and stability, were formulated. OT and its radiolabeled analogue [^14^C]‐OT were synthesized in high yields, enabling in vivo pharmacokinetic studies in mice. Results demonstrated significantly higher brain uptake and reduced systemic exposure of OT‐NP‐PEG via IN administration compared to OT in solution. Preliminary behavioral findings suggest central engagement of OT signaling, warranting further research in disease‐relevant models. Collectively, these results advance the field of NTB delivery of OT and highlight the potential of nanoparticle‐based formulations to improve therapeutic outcomes in OT‐associated neurological and psychiatric disorders.

## 5 Experimental Section

### Materials

Poly (lactic‐co‐glycolic acid) 17K (LA:GA 50:50) and PLGA (17K)‐PEG‐COOH,3.4K (LA:GA: 50:50) were obtained from Purac Biomaterials and Biochempeg, respectively. Soybean lecithin (EpikuronTM 145) was supplied by Cargill. Oxytocin was purchased from Fluorochem. Porcine stomach Type II mucin, DNA (Calf Thymus), Tween 80, calcium chloride anhydrous, potassium chloride, trifluoroacetic acid, MTT, diisopropylethylamine, di‐tert‐butyl decarbonate, triethyl silane were supplied by Sigma‐Aldrich. Phenobarbital sodium was obtained from Boehringer Ingelheim. Isoflurane (IsoFlo) was obtained from Abbott laboratories Ltd. Egg yolk tellurite emulsion was procured from VWR Chemicals. Transwell Inserts were supplied by Corning Inc. Corning 12‐well plates were obtained from Becton Dickinson. Micro BCA Kit, Dil fluorescent dye was purchased from Thermo scientific. UA‐zero stain was purchased from Agar scientific. Fetal bovine serum, penicillin/streptomycin, and Trypsin/EDTA, Sodium GlutaMAX, sodium pyruvate, MEM and phosphate‐buffered saline were purchased from Thermo Fisher. TentaGel R Ram was obtained from Rapp Polymere. Fmoc‐Glycine,[1‐^14^C] was purchased from Vitrax USA. Fmoc‐amino acids were purchased from Novbiochem. O‐(1H‐6‐Chlorobenzotriazole‐1‐yl)‐1,1,3,3‐tetramethyluronium hexafluorophosphateO‐(1H‐6‐Chlorobenzotriazole‐1‐yl)‐1,1,3,3‐tetramethyluronium hexafluorophosphate (HCTU), piperidine, and acetic acid (glacial) were supplied by Merck Millipore. N, N’‐Diisopropylcarbodiimide was obtained from Honeywell Fluka. Picryl sulfonic Acid, N, N’ diisopropylethylamine were purchased from TCI. Dimethylformamide was supplied by Acros Organics. Anhydrous DMSO was purchased from Biotium. Isopropanol, hydrogen peroxide, and 2,2*′‐*dithiodipyridine were obtained from Fisher Scientific. Solvable was purchased from Perkin Elmer. Scintilogic^TM^ U was supplied by Lab Logic Systems.

### Methods: Formulation of Oxytocin‐Loaded PLGA (OT‐NP) and PLGA‐PEG Nanoparticles (OT‐NP‐PEG)

OT‐NP and OT‐NP‐PEG were formulated by the nanoprecipitation method using optimized process parameters. Briefly, OT was dissolved in the aqueous phase containing Tween 80 (w/v). The organic phase, consisting of PLGA or PLGA‐PEG and soy lecithin (w/v) in acetone/ethanol (v/v), was sonicated and vortexed to ensure complete dissolution. It was then added dropwise to the aqueous phase under continuous stirring at 2000 rpm for 3 h to allow gradual solvent evaporation and the formation of NPs. The final volume was adjusted to 5 mL with deionized water. The nanoparticle (NP) suspension (5 mL) was transferred to OptiSeal centrifuge tubes, diluted with deionized water to a final volume of 8.9 mL, and ultracentrifuged at 35000 rpm for 60 min at 4 °C. The supernatant containing unencapsulated OT was discarded, and the pellet was collected and resuspended in 5 mL deionized water by brief sonication and vortexing to obtain a homogeneous nanosuspension for physicochemical characterization. For cytotoxicity studies, pellets were similarly resuspended in culture media.

### Methods: Experimental Design and Construction of 3‐Level Factorial Design

Based on preliminary experiments, a 3^3^ full factorial design was used to optimize OT‐NP and study the main, quadratic, and interaction effects on PS, polydispersity index (PDI), and DL%. Three preparation variables; polymer concentration (X1), lecithin concentration (X2), and Tween 80 concentration (X3) were evaluated at low, medium, and high levels across 27 combinations. OT amount (4 mg), organic‐to‐aqueous phase ratio (1:2), and stirring speed (2000 rpm) were kept constant. The experimental responses (Y1: PS, Y2: PDI, Y3: DL%) were analyzed using Design Expert software (version 12.0). Regression, polynomial model fitting, and analysis of variance (ANOVA) were used to evaluate variable effects and interactions (X1X2, X1X3, X2X3) via the following polynomial equation

(4) $$\begin{array}{c} Y=b0+B1X1+B2X2+B3X3+B4\mathrm{XIX}2+B5X1X3+B6X2X3+\\ B7X12+B8X22+B9XX2 \end{array}$$

where *y* is the dependent variable, *b*_0_ is the intercept, and *b*_1_ to *b_9_* are the regression coefficients. X_1_, X_2_ and X_3_ are the coded values of the independent variables. X_a_ X_b_ (a,b = 1, 2, 3) and Xi^2^ (i = 1,2,3) represent the interaction and quadratic terms, respectively. The three‐dimensional response surface and contour plots for PS (Y1), PDI (Y2), and DL% (Y3) were plotted according to the regression model with one variable fixed at the center level.

### Methods: Characterization of OT Loaded NPs

*Determination of particle size distribution and zeta potential*: The PS, PDI and zeta potential of the NPs were measured using a Malvern NanoZS (UK). PS and PDI were determined in polystyrene cuvettes, and zeta potential was determined in capillary cells. NP suspensions were diluted 1:100 in deionized water, and measurements were performed in triplicate at 25 °C. Zeta potential was calculated from electrophoretic mobility using the Helmholtz–Smoluchowski equation. Results are reported as mean ± SD.

*Determination of the drug loading capacity (DL%)*: The DL% was determined from ultracentrifuged OT‐NP and OT‐NP‐PEG pellets (35 000 rpm, 4 °C, 60 min), which were dissolved in dichloromethane: ethanol (7:3) and then dissolved by sonication and vortexing. OT was extracted with 1 mL HPLC‐grade water, centrifuged (14 800 rpm, 4 °C, 10 min), and filtered (0.2 µm). OT content was quantified by RP‐HPLC (Agilent Poroshell 120 EC‐C18, 40 °C) using a water/acetonitrile‐ trifluoroacetic acid (TFA) gradient, detected at 220 nm. A calibration curve (7.8–600 µg mL^−1^ OT) with vasopressin (20 µg mL^−1^) as an internal standard was used to calculate DL%.

(5) $$\mathrm{DL}\%=\left(\frac{\mathrm{Amount}\;\mathrm{of}\;\mathrm{OT}\;\mathrm{encapsulated}}{\mathrm{Total}\;\mathrm{weight}\;\mathrm{of}\;\mathrm{nanoparticles}}\right)\times \;100$$

*Transmission electron microscopy imaging (TEM)*: For TEM, a drop (≈5 µL) of NP dispersion (1 mg mL^−1^) was deposited on a carbon‐coated 300‐mesh copper grid (Agar Scientific Ltd, UK) and allowed to stand for 3 min, after which filter paper absorbed any excess fluid. One cycle of quick washing with filtered deionized water was performed, and the sample was left to air‐dry. NPs were stained with a drop of uranyl acetate‐Zero (Agar Scientific Ltd, UK) for 3 min, and any excess fluid was then absorbed by filter paper and left to air‐dry before imaging. An FEI Tecnai T12 G2 Spirit with an 11‐megapixel Olympus side mount morada camera was used for imaging at 80 kV.

*Nanoparticle tracking analysis (NTA)*: The size distribution, particle number and concentration of NPs were measured by NTA using a NanoSight LM10 system with a blue (488 nm) laser (Malvern Instruments, UK). NPs were diluted 5k times in filtered deionized water to obtain 20–80 particles per field of view for optimal tracking. The modal size and particle count were measured in triplicate, with 30 s as the duration for each recording, and analyzed using the NanoSight NTA 3.2 software. All NTA measurements were performed with identical system settings for consistency.

*Shelf‐life stability*: The OT‐NP or OT‐NP‐PEG dispersions were sealed in transparent 7 mL glass vials and stored at 4 °C. The stability of the NP dispersions was tested at 0, 7, 14, and 28 days by visual inspection of the physical properties (color and opacity) and measurement of PS, zeta potential, and PDI. The measurements were performed in triplicate and presented as mean ± SD.

*In vitro release study*: The OT‐NP or OT‐NP‐PEG were suspended in deionized water and dialyzed (10 kDa molecular weight cut‐off) against phosphate‐buffered saline (PBS) at pH 7.4, at a shaking rate of 100 stroke min^−1^ using a shaking incubator (SciQuip Incu‐Shake MAXI, UK) for 48 h. Samples were withdrawn at predetermined time intervals (0.5, 1, 2, 4, 6, 24, and 48 h). To maintain sink conditions, the withdrawn samples were replaced with an equal volume of the release medium. The OT concentration in the dialysate was quantified by RP‐HPLC using a calibration curve for OT in PBS (pH 7.4). To eliminate nonspecific drug adsorption to the dialysis membrane, free OT dissolved in deionized water was dialyzed against the release media for comparison.

### Cellular Studies: Cell Culture

RPMI 2650 cells were cultured in Minimum Essential Media Eagle (MEM)O‐(1H‐6‐Chlorobenzotriazole‐1‐yl)‐1,1,3,3‐tetramethyluronium hexafluorophosphate (M4655) medium supplemented with 10% FBS, 1% penicillin/streptomycin, 1% non‐essential amino acid and 1% sodium pyruvate. The cells were cultured in a 75 cm^2^ flask under standard conditions and incubated at 37 °C with 5% CO_2_ with medium changes performed thrice weekly. The cells were detached for passaging when confluence reached ≈80% by treating the cells with trypsin‐EDTA at 37 °C for 5 min. The cells were centrifuged at 200 × g for 5 min at room temperature. The cell pellet was then resuspended in the culture medium and counted with a heemocytometer. The cells were seeded into new flasks at a 1:3 split ratio.

### Cellular Studies: Cell Viability Study

Cell viability was assessed using the 3‐(4,5‐dimethylthiazol‐2‐yl)‐2,5‐diphenyltetrazolium (MTT) assay. Cells were seeded at 2 × 10⁴ cells per well and incubated overnight. OT‐NP or blank NP pellets were resuspended in culture media to prepare serial concentrations (0.25–4 mg mL^−1^ PLGA; 6–96 µg mL^−1^ OT). Cells were treated with 100 µL of NP suspension for 72 h. The media was then removed, and 120 µL MTT reagent was added for 3 h at 37 °C. Formazan crystals were dissolved in 200 µL DMSO and incubated for 5 min at 37 °C. Absorbance was measured at 570 nm (FLUOstar OPTIMA, BMG Labtech, Germany), and cell viability (%) was calculated and expressed as mean ± SD (n = 5).

(6) $$\%\;\mathrm{Cell}\;\mathrm{survival}\;=\left(\frac{A\;570\;\mathrm{nm}\;\mathrm{treated}\;\mathrm{cells}}{A\;570\;\mathrm{nm}\;\mathrm{untreated}\;\mathrm{control}\;\mathrm{cells}}\right)\times \;100$$

### Characterization of OT by RP‐HPLC and LC‐MS

The synthesized linear and cyclic purified OT peptides were purified and analyzed using RP‐HPLC on an Agilent Poroshell 120 EC‐C18 column (2.1 × 50 mm, 2.7 µm) with a C18 guard column. The mobile phase consisted of water/acetonitrile (95:5 v/v) with 0.05% trifluoroacetic acid (TFA) (A) and acetonitrile/water (95:5 v/v) with 0.025% TFA (B). Samples were prepared in HPLC‐grade water and injected via an autosampler, with UV detection at 220 nm. Quantification was performed using a standard calibration curve of commercial OT (10–500 µg mL^−1^), and the purified yield was calculated from RP‐HPLC results. LC‐MS analysis was performed on an Agilent 6120 Quadrupole system using an Agilent Zorbax Eclipse Plus C18 RRHD column (1.8 µm, 2.1 × 50 mm, 95 Å) to monitor oxidation and confirm the identity of synthesized OT. The mobile phase consisted of water and acetonitrile, and a gradient was applied from 10% to 90% acetonitrile over 8.5 min, followed by re‐equilibration to 10% Acetonitrile over 0.5 min, with a total run time of 10 min. The flow rate was 0.5 mL min^−1^, injection volume was 10 µL, and the UV detection range was 220–400 nm.

### Synthesis of [^14^C] OT

The [^14^C] OT was synthesized using the Fmoc‐based SPPS. A 500 µCi of Fmoc‐Gly [1‐^14^C] in acetonitrile was purchased from Vitrax, USA, as the starting amino acid residue for synthesizing [^14^C] OT. The acetonitrile containing Fmoc‐Gly [1‐^14^C] from the shipped hot vial was transferred to an Eppendorf vial and gently evaporated the acetonitrile using a slow nitrogen flash. The hot vial was washed multiple times with fresh acetonitrile and repeated the same step until all the radioactivity (RA) was transferred to the Eppendorf vial. A 10 µL aliquot was taken from the hot sample and added to a scintillation vial containing 4 mL of scintillation cocktail (containing 0.7% glacial acetic acid). The sample was subjected to a liquid scintillation counter (LSC) (Beckman Coulter LS 6500 Auto counter, Beckman Coulter Life Sciences, (USA) to determine the total RA transferred and the remaining RA in the hot vial.

The coupling procedure of Fmoc‐Gly [1‐^14^C] was carried out stepwise to couple a 5‐fold molar excess of Fmoc‐Gly, including Fmoc‐Gly[1‐^14^C], to the resin. Initially, a 1‐fold excess coupling was carried out by combining Fmoc‐Gly [1‐^14^C] (hot) and cold Fmoc‐Gly. Then, the remaining 4 mole excess cold Fmoc‐Gly was coupled to the resin. Briefly, the dried 2.5 mg of Fmoc‐Gly [1‐^14^C] was activated in 28 µL anhydrous DMSO containing 3.3 mg HCTU and 2.85 µL of DIPEA in an Eppendorf vial followed by gentle pipetting to dissolve the mixture completely. The swollen resin in DMF (deprotected) was added to the hot Eppendorf vial and subjected to a coupling reaction using a rotator for 1 h. After 1 h of coupling reaction, the amount of RA in the supernatant was determined by taking a 10 µL of the solvent and indirectly calculated the amount of RA coupled to the resin beads, compared to the initial amount of RA added. In the case of high RA in the supernatant compared to the resin beads, the coupling reaction was further proceeded until maximum RA was coupled to the resin as determined by liquid scintillation counting (LSC). A 12.36 mg cold Fmoc‐Gly in 139 µL Anhydrous DMSO containing 16.3 mg HCTU and 14.86 µL DIPEA was added and subjected to coupling reaction for 1 h. The success of the coupling reaction was confirmed using the TNBS test. Chain extension was carried out by coupling the subsequent Fmoc‐amino acid residues to the growing peptide on resin using standard SPPS. The RA of the filtrate after each coupling reaction was determined using LSC to ensure complete reactions. The resin was washed with DMF followed by the Fmoc deprotection using 20% piperidine in DMF. The weight and total RA of the crude linear [^14^C] OT were determined to calculate radiochemical yield (SI). Cyclisation and purification were subsequently carried out, followed by freeze‐drying the peptide in aliquots.

### In Vivo Studies: Animals

All the animal experiments were performed under the authority of project and personal licenses granted by the UK Home Office and in compliance with the UK Home Office (1986) Code of Practice for the Housing and Care of Animals Used in Scientific Procedures. Male and female CD‐1 mice (18–25 g, 4–6 weeks old) were used for in vivo organ biodistribution studies. Female C57BL/6 mice (18–25 g, 4–6 weeks old) were used for in vivo behavioral study. All mice were obtained from Charles River (UK).

### In Vivo Studies: In Vivo Dosing and Sample Preparation for Organ Biodistribution Study

A dose of 0.1 µCi/20 µL (22.4 µg OT) per mouse was prepared from OT‐NP‐PEG for IN administration, and 0.1 µCi/100 µL for IV administration in sterile PBS. Six‐ to eight‐week‐old CD‐1 mice received IN doses (10 µL per nostril, administered over 2–3 min) or IV doses via the tail vein under isoflurane anesthesia. At 10, 30, 60, and 1440 min, mice were terminally anaesthetized (IP sodium pentobarbital, 50–90 mg kg^−1^), and blood was collected from the inferior vena cava. Major tissues (nasal cavity, brain, heart, lung, liver, spleen, stomach, intestine, and kidney) were excised, weighed, and processed for LSC, with nasal, stomach, and intestine mechanically chopped. Reference tissues were prepared for normalization and quenching assessment. Reference tissue samples (cold) were prepared in a similar manner for normalization and to investigate any quenching effect by the sample tissues.

### In Vivo Studies: Translocation of [^14^C] OT and [^14^C] OT‐NP‐PEG in Brain Regions

In this experiment, CD‐1 mice were intranasally administered under inhalational anesthesia with formulations by dosing 2 µL to the left and right nostrils alternatively at a minimum interval of 20 s. A total of 20 µL was administered to each mouse. At 10 and 60 min, the mice were euthanized under terminal anesthesia followed by cervical dislocation. The whole brain was carefully collected and spatiotemporally dissected into 4 sections: Olfactory lobe, cerebrum 1, cerebrum 2, cerebellum plus brain stem. Each brain section was weighed using a digital weight balance (Sartorius Secura, scientific laboratory supplies, UK) and processed for LSC.

### In Vivo Studies: Sample Processing for Liquid Scintillation Counting

The sample tissues were digested in 1–3 mL Solvable at 55 °C for 12–24 h, then bleached with H_2_O_2_ until the greenish color disappeared. Isopropanol was added to remove bubbles, and samples were incubated at 55 °C for 3 h to eliminate residual H_2_O_2_. Each sample was mixed with 10 mL scintillation cocktail and stored overnight in the dark. Reference doses (0%, 1%, 2.5%, 5%, 10%, 100% IDg^−1^) were prepared for each experiment. Samples were analyzed by LSC, and results expressed as % ID g^−1^.

### In Vivo Studies: Self‐Grooming Behavioral Study of OT‐NP‐PEG in Mice

Mice received IN OT‐NP‐PEG (0.7 mg kg^−1^ OT) or blank NP in 5 µL sterile PBS without anesthesia. Self‐grooming behavior was assessed in a clean, empty arena (46 × 23.5 × 20 cm) with a transparent plexiglass cover, 20 lux illumination, and video recording from 1 meter above. Mice were acclimatized to the behavioral room for 30 min, then filmed for 15 min, including a 5 min habituation period. Self‐grooming behavior over 10 min was scored from the recordings, with all treatments and analyses performed blind to the researcher.

### Statistical Analysis

Data for OT‐NP and OT‐NP‐PEG are presented as mean ± SD (n = independent replicates). Release and diffusion studies were analyzed using two‐way ANOVA with Sidak's multiple comparisons test. In vivo organ biodistribution data are shown as mean ± SEM (n = replicates) and analyzed by one‐way ANOVA with Tukey's post hoc test. Mice behavioral data are expressed as mean ± SEM (n = 12) and analyzed using unpaired Student's *t*‐test. Statistical significance was defined as *p < 0.05, **p < 0.01, ***p < 0.001, ****p < 0.0001.

## Conflict of Interest

The authors declare no conflict of interest.

## Supporting information



Supporting Information

## Data Availability

The data that support the findings of this study are available in the supplementary material of this article.
