# Supplementary material for: Design of Experiments (DoE)‐Optimized Polymeric Oxytocin Nanoparticles for Enhanced Nose‐to‐Brain Delivery
Source: Small. 2025 Dec 19;22(8):e11603. doi: 10.1002/smll.202511603 (PMC12877978; doi:10.1002/smll.202511603)
Supplement: Supplementary file 1 — Supporting Information [file SMLL-22-e11603-s001.pdf]

NANO · MICRO
small

Supporting Information

for *Small*, DOI 10.1002/smll.202511603

Design of Experiments (DoE)-Optimized Polymeric Oxytocin Nanoparticles for Enhanced Nose-to-Brain Delivery

*Naveed Ahmad, Shunping Han, Rifka Utami, Rafal Baker, Dina Helal, Zhuoni Li, Mark Tricklebank, Yannis Paloyelis, Julie Wang, Marija M. Petrinovic, Sukhi Bansal and Khuloud T. Al-Jamal**

Design of Experiments (DoE)-Optimized Polymeric Oxytocin Nanoparticles for Enhanced Nose-to-Brain Delivery

Naveed Ahmad[1], Shunping Han[1], Rifka Utami[1,2], Rafal Baker[1], Dina Helal[1,3], Zhuoni Li[4], Mark Tricklebank[5], Yannis Paloyelis[5], Julie Wang[1], Marija M. Petrinovic[4,5,6], Sukhi Bansal[1], and Khuloud T. Al-Jamal[1,7] *

1. Institute of Pharmaceutical Science, King's College London, Franklin-Wilkins Building, 150 Stamford Street, London SE1 9NH, UK
2. Faculty of Pharmacy, Hasanuddin University, 90245 Makassar, Indonesia
3. Department of Pharmaceutics and Industrial Pharmacy, Faculty of pharmacy, Ain Shams University, Monazzamet El Wehda El Afrikeya Street, Abbasseya, Cairo, Egypt
4. Department of Forensic and Neurodevelopmental Sciences, Institute of Psychiatry, Psychology and Neuroscience, King's College London, 16 De Crespigny Park, London SE5 8AF, UK
5. Department of Neuroimaging, Institute of Psychiatry, Psychology and Neuroscience, King's College London, De Crespigny Park, London SE5 8AF, UK
6. Medical Research Council Centre for Neurodevelopmental Disorders, King's College London, New Hunt's House, London SE1 1UL, UK
7. Department of Pharmacology and Pharmacy, Li Ka Shing Faculty of Medicine, The University of Hong Kong, Hong Kong Special Administrative Region, China

* Corresponding authors

E-mail: khuloud.al-jamal@kcl.ac.uk; kaljamal@hku.hk

Keywords: artificial intelligence , autism, autoradiography, encapsulation, peptide, PLGA, radiolabelling, .

Supplementary Methods

Methods

Preparation of simulated nasal fluid and mucus

The simulated nasal fluid (SNF) was prepared from sodium chloride (0.877 %), calcium chloride (0.058 % *w*/*v*), and potassium chloride (0.298 % *w*/*v*) dissolved in deionized water and adjusted the pH to 6.8 [1]. For preparation of simulated nasal media (SNM), the porcine mucin-type II was added to a buffer solution containing 7.5 mg/mL of sodium chloride, 1.3 mg/mL of potassium chloride, and 0.3 mg/mL of calcium chloride dihydrate. This resulted an overall concentration of porcine mucin of 8 % (m/m) in buffer solution [2]. The pH was adjusted to 7.0 using a NaOH solution.

Fluorescence labelling of OT-NP and OT-NP-PEG

The OT-NP and OT-NP-PEG were labelled with the hydrophobic dye, 1,10 - dioctadecyl-3,3,30 30 -tetramethyl indocarbocyanine perchlorate (DiI) using the nanoprecipitation method. Briefly, the DiI (0.5 % w/w of polymer) was dissolved in an organic phase containing PLGA or PLGA-PEG (6 mg/mL) and soy lecithin (0.3 % w/v). The organic phase was added dropwise to the aqueous phase containing OT (4 mg) and Tween® 80 (0.1 % w/v) and formulated as described earlier. The nanosuspension was subjected to gel filtration to remove the unentrapped dye using a pre-equilibrated PD-10 desalting column, bed volume 8.3 mL (GE Healthcare, USA). Briefly, 2.5 mL of OT-NP or OT-NP-PEG suspension was added to the PD-10 column and allowed to move freely in the column bed by gravity force. The NPs were eluted by adding deionized water (1 mL per run) until the recovery in 3-4 fractions. The method was previously optimized using unlabeled NPs. The elution fractions of DiI-loaded OT-NP or OT-NP-PEG were combined and concentrated (10x) under reduced pressure using a rotary evaporator (Rotavapor® R-210, Buchi UK). To evaluate the amount of loaded dye, 1.5 mL DMSO was added to break the NPs. A 90 µL sample of dye-loaded OT-NP or OT-NP-PEG with 10 µL ethanol was added to 96-well plate (n=5). The plate was read by a plate reader (FLUOStar Omega) at excitation/emission = 549/565 nm. To make a standard calibration curve, the same experimental conditions were applied for unlabeled OT-NP or OT-NP-PEG which were then mixed with a known amount of DiI (equivalent to 0.78-100 % dye encapsulation) in a 96-well plate. The dye encapsulation efficiency was calculated using the following formula.

$$\text{Dye Entrapment efficiency (\%)} = \left(\frac{\text{Amount of dye in yielded NPs}}{\text{Amount of dye added to formulation}}\right) \times 100$$

Mucin diffusion study

A Corning® 12-well plate containing Transwell® inserts was used as a diffusion cell setup for mucin diffusion study. A commercial bicinchoninic acid (BCA) assay kit (Thermo Fisher Scientific UK) was used to determine the optimum range of time intervals for the diffusion of fluorescently labelled OT-NP or OT-NP-PEG through SNM. Briefly, 200 µL of SNM was added to a membrane of Transwell® inserts and placed in a 12-well plate filled with 1 mL simulated nasal fluid (SNF). A sample of 25 µL from the basolateral compartment was taken at various time intervals (0, 5, 10, 20, 30, 40, 60, 120, 180, 240, 1440 min) and added to 96 wells plate. A 200 µL working solution (BCA) was added to each well, shaken for 30 sec, and incubated at 37 °C for 30 min. The absorbance values were measured using a plate reader at 562 nm. A calibration curve (equivalent to 0.78-100 % mucin diffusion) was constructed using the same experimental conditions without Transwell® inserts and measured by a plate reader at 562 nm. The % mucin diffusion was calculated against the calibration curve. The measurements were performed in triplicate and presented as mean ± SD.

Effect of SNM on physicochemical properties of OT-NP and OT-NP-PEG

The effect of SNM on particle size, PDI or zeta potential of OT-NP or OT-NP-PEG was studied. NP suspension and mucus were mixed at a 1:1 volume ratio and incubated at 37°C at different intervals [3]. Briefly, a 200 µL sample of 10x concentrated OT-NP or OT-NP-PEG was mixed with 200 µL SNM in an Eppendorf™ tube and incubated for 24 h at 37 °C. A 10 µL sample was taken at 5, 10, 60, 120, 240 min and 24 h and diluted 1000 times. The particle size, PDI and zeta potential distribution were measured using the Helmholtz–Smoluchowski equation and compared with the control group using Two-way ANOVA followed by Dunnett's multiple comparison test.

Diffusion of fluorescently labelled OT-NP and OT-NP-PEG through SNM

The fluorescently (DiI) labelled loaded OT-NP or OT-NP-PEG was prepared using the nanoprecipitation method. The diffusion OT-NP or OT-NP-PEG in SNM was conducted using Transwell® inserts. Briefly, 200 µL of DiI-loaded OT-NP or OT-NP-PEG was added to the upper compartment of Transwell® inserts containing 200 µL of SNM and kept at room temperature. A sample of 25 µL was taken from the basolateral compartment at a pre-determined time point (0, 5, 10, 20, 30, 40, 50, 60, 120, 180, 240 min) and added to 96 well-plat. A 75 µL of DMSO was added to each well to break the NP. A standard calibration curve was constructed using the same experimental condition. Briefly, a 200 µL DiI loaded OT-NP or OT-NP-PEG was added to 1.5 mL Eppendorf™ containing 1 mL SNF and SNM equivalent to 100% diffusion. Further dilutions were prepared equivalent to 50, 25, 12.5, 6.25, 3.1 and 1.5 % diffusion. A 25 µL sample was taken from each dilution and added to 96-well plate with 75 µL DMSO. The amount of NP permeated was evaluated by monitoring the fluorescence intensity (excitation/emission 544/590 nm) using plate reader. The % diffusion of OT-NP or PEG OT-NP was calculated against the calibration curve using two-way ANOVA followed by Sidak's multiple comparison test. Measurements were performed in triplicate and presented as an average ± SD.

In vivo dose-optimization study

CD-1 mice were used for dose optimisation *in vivo*. A dose of 60 or 180 µg of commercial OT was prepared in 20 µL of 0.9 % normal saline solution. The body weight of each mouse was recorded before the dose administration. Mice were administered IN with 60 or 180 µg of OT in 20 µL (10 µL per nostril) under inhalation anaesthesia. The mice were transferred into individual cages for close observation of any aggressive behaviour, seizure, loss of balance and sedation up to 5 h. The body weight of each mouse was recorded at 1, 2, 3, 4, 5, 24 h and day 5 of dose administration. The % change in body weight was calculated and presented.

Synthesis of OT and [^{14}C] OT by solid phase peptide synthesis (SPPS)

OT was synthesised by SPPS using fluorenyl methoxycarbonyl (Fmoc) chemistry on TentaGel® resin (0.2 mmol/g). Amino acids were coupled sequentially with HCTU/DIPEA in DMF, and Fmoc groups were removed with 20% piperidine in DMF. The peptide sequence Gly-Leu-Pro-Cys (Trt)-Asn(Trt)-Gln (Trt)-Ile-Tyr(tBu)-Cys(Trt) was built on resin and capped with Boc2O. Cleavage from the resin and global sidechain deprotection were achieved with a TFA-based cocktail for 2 h. The crude peptide was precipitated with cold ether, centrifuged, dried under nitrogen, and lyophilised. The linear peptide was then cyclised in Tris-HCl buffer (pH 8.0) with 2,2′-dithiodipyridine (2-PDS) for 2 h, and oxidation was confirmed by RP-HPLC and LC-MS. Finally, the cyclic peptide was purified on a C18 SPE column, dried, redissolved in HPLC-grade water, and freeze-dried for characterisation.

Characterization of [^{14}C] OT by RP-HPLC

The radioactive OT samples (5, 10, 15, and 20 µg/mL) were prepared based on mock HPLC analysis of hot OT in the designated radio lab per the approved protocols. The radioactive samples were added to HPLC glass vial inserts, closed with caps, and double-protected using a vial rack. The samples were brought to the designated RP-HPLC area and put in the autosampler. The column was pre-washed using the established method. A blank sample was run to ensure a straight baseline was obtained. A standard calibration curve (2.5-40 µg/mL) was constructed using commercial cold OT. The [^{14}C] OT samples were analysed by RP-HPLC using the established protocols. The fraction of OT peak in each sample concentration was collected in the pre-labelled vials with a 3 min retention time interval while the remaining waste was collected in a separate vial. At the end of the operation, the column was washed, and the designated surfaces were checked for contamination using LSC.

Quantification of [^{14}C] by LSC

The fraction of OT peak in each sample was added to 4 mL scintillation vial containing a scintillation cocktail (ScintLogic^{TM} U) and subjected to liquid scintillation counting to measure the amount of radioactivity. A standard calibration curve was obtained from

the RA values of each hot OT fraction. The total RA yield was calculated from the calibration curve. The waste vials were also measured by LSC.

Self-grooming behavioural study of free OT after IP administration in mice

Mice received IP injections of Vehicle (10 mL/kg sterile PBS) or OT (50 mg/kg) to assess the frequency of self-grooming behaviour (number of bouts). OT and Vehicle were administered in a volume of 10 mL/kg. Self-grooming behaviour was evaluated in a clean, empty chamber (46 × 23.5 × 20 cm) with a transparent plexiglass cover under 20 lux illumination. Behaviour was video recorded from 1 meter above. Mice were acclimatized in the behaviour room for 30 minutes before testing and then filmed for 15 minutes, including a 5-minute habituation period. Self-grooming during the subsequent 10 minutes was scored from the recordings. All treatments and analyses were performed with the experimenter blinded to group assignments.

Supplementary Results

Fluorescence labelling of OT-NP and OT-NP-PEG

The OT-NP and OT-NP-PEG were labelled with the hydrophobic dye (DiI), incorporated in the organic phase. Rapid precipitation of the polymer with the fluorescent dye is necessary for effective encapsulation because it confines the dye inside the polymer matrix and prevents its diffusion to the aqueous phase. The dye encapsulation efficiency was determined using a standard calibration curve for OT-NP (R^2 = 0.993) and OT-NP-PEG (R^2 = 0.990). The dye encapsulation efficiency for OT-NP and OT-NP-PEG was 78.34 % and 84.22 %, respectively. The encapsulation of DiI did not produce any considerable changes in the physicochemical properties of the OT-NP and OT-NP-PEG (**Table S10**). The DiI-loaded OT-NP and OT-NP-PEG had a mean diameter of 114.25 nm and 107.6 nm, respectively. Similarly, the PDI was low (~ 0.2) and exhibited negative zeta potentials by both NPs.

Effect of SNM on physicochemical properties of OT-NP and OT-NP-PEG

OT-NP showed no significant change in size after exposure to SNM from 0 to 240 min (**Figure S7**). However, the size distribution after 24 h of incubation with SNM increased from 108.81 nm to 156.64 nm. It showed a change in the appearance of NP peaks, most probably attributed to mucin and mucin/NP aggregates. A similar trend of change in particle size was observed for OT-NP-PEG after 24 h, and the PS increased from 91.48 nm to 117.50 nm. These results suggest that mucin may be able to bind or immobilise on the surface of OT-NP or OT-NP-PEG if incubated for a longer period. Additionally, the degree of mucin protein interaction between particles may increase as mucin binds to the particles, rather than only mucin coating on individual particles [4]. The PDI of OT-NP was in an acceptable range (~ 0.2) at 5-, 10-, and 60-min incubation with SNM. It increased to 0.32 and 0.37 at 240 min and 1440 min, respectively. This indicated a wider range of particle size distribution due to the binding or immobilising of mucin on the surfaces of OT-NP. However, the OT-NP-PEG showed no significant increase in PDI during the study period. This is because the hydrophilic PEG coating on OT-NP may create a hydrated cloud which can sterically prevent the NP from interacting with mucin. [5]. It is imperative that both OT-NP and OT-NP-PEG retain their zeta potential values, and no significant changes have been observed during the study period. The zeta potential of OT-NP at 1, 4 and 24 hr were found to be -28.60, -23.46, and -17.61 mV, respectively. For OT-NP-PEG, the zeta potential values were well preserved (-19.8 to -18.1).

Table S1. Effect of preliminary formulation process parameters on NP physico-chemical properties.

Formulation code [a]	Stirring speed	Organic solvent (v/v)	Lecithin (% w/v)	Tween™ 80 (% w/v)	Size (nm) [b, c]	PDI [2,3]	Zeta potential (mV) [c, d]
**Stirring speed**							
NP-1	1000	ACT+ EtOH (60:40)	0.5	0.2	147.8 ± 7.6	0.29 ± 0.02	-15.4 ± 2.3
NP-2	1500	ACT+ EtOH (60:40)			131.9 ± 5.0	0.21 ± 0.07	-18.9 ± 1.5
NP-3	2000	ACT+ EtOH (60:40)			112.9 ± 2.7	0.18 ± 0.01	-20.5 ± 1.7
**Organic solvent**							
NP-4	2000	ACT+ EtOH (60:40)	0.5	0.2	113.3 ± 2.7	0.18 ± 0.01	-19.8 ± 1.1
NP-5		ACN+ EtOH (60:40)			137.9 ± 3.2	0.27 ± 0.02	-15.8 ± 1.4
NP-6		ACT+ EtOH (80:20)			96.6 ± 3.4	0.16 ± 0.01	-21.6 ± 1.9
NP-7		ACN+ EtOH (80:20)			127.8 ± 5.2	0.18 ± 0.01	-17.1 ± 1.4
**Lecithin concentration**							
NP-8	2000	ACT+ EtOH (80:20)	0.3	0.2	86.7 ± 1.7	0.16 ± 0.04	-22.4 ± 2.2
NP-9			0.5		96.5 ± 3.4	0.15 ± 0.01	-19.3 ± 1.4
NP-10			0.6		142.2 ± 6.2	0.30 ± 0.02	-13.5 ± 1.0
**Tween® 80 concentration**							
NP-11	2000	ACT+ EtOH (80:20)	0.3	0.1	131.5 ± 4.2	0.26 ± 0.02	-18.3 ± 2.0
NP-12				0.2	85.8 ± 2.5	0.16 ± 0.04	-22.0 ± 1.8
NP-13				0.3	79.1 ± 1.3	0.14 ± 0.07	-36.9 ± 2.9

[a] Total PLGA 15 mg, organic to aqueous volume ratio 1:2, organic volume 2.5 mL, aqueous volume 5 mL
[b] Measured by dynamic light scattering
[c] Expressed as mean ± SD (n=3)
[d] Zeta potential, calculated by electrophoretic mobility

Table S2. Experimental design, parameters and responses for 3^3 factorial design of OT-NP.

S.No	Batch # [a]	PLGA (mg/ml)	Lecithin (%w/v)	Tween® 80 (% w/v)	Size (nm)[b,d]	PDI [b,d]	% DL[c,d]
**1**	F1	8	0.5	0.3	167.32	0.316	1.48
**2**	F2	8	0.3	0.1	152.32	0.281	2.06
**3**	F3	6	0.1	0.2	92.11	0.180	1.81
**4**	F4	8	0.1	0.2	123.74	0.280	1.26
**5**	F5	4	0.1	0.1	110.63	0.261	1.40
**6**	F6	4	0.5	0.1	135.78	0.282	2.06
**7**	F7	4	0.3	0.2	141.42	0.291	2.12
**8**	F8	8	0.1	0.1	131.64	0.233	0.76
**9**	F9	4	0.3	0.3	125.69	0.202	1.64
**10**	F10	6	0.5	0.2	136.44	0.281	2.61
**11**	F11	4	0.5	0.1	139.54	0.341	2.12
**12**	F12	6	0.3	0.1	116.21	0.182	2.76
**13**	F13	8	0.5	0.1	159.12	0.341	2.22
**14**	F14	6	0.1	0.2	111.39	0.242	1.49
**15**	F15	8	0.3	0.3	143.66	0231	1.36
**16**	F16	8	0.5	0.3	165.34	0.370	2.33
**17**	F17	6	0.3	0.2	97.91	0.168	2.53
**18**	F18	6	0.5	0.2	126.42	0.263	1.94
**19**	F19	8	0.1	0.1	134.22	0.320	1.44
**20**	F20	6	0.1	0.3	98.49	0.690	1.12
**21**	F21	6	0.5	0.3	133.12	0.234	1.45
**22**	F22	4	0.1	0.2	91.52	0.216	1.11
**23**	F23	4	0.3	0.3	120.12	0.213	1.36
**24**	F24	6	0.3	0.3	108.14	0.174	1.75
**25**	F25	4	0.1	0.3	93.66	0.169	0.96
**26**	F26	8	0.3	0.2	139.31	0.261	1.86
**27**	F27	4	0.5	0.3	141.76	0.294	0.90

[a] Amount of OT is 4 mg
[b] Measured by dynamic light scattering
[c] Quantified by RP-HPLC
[d] Expressed as mean ± SD (n=3)

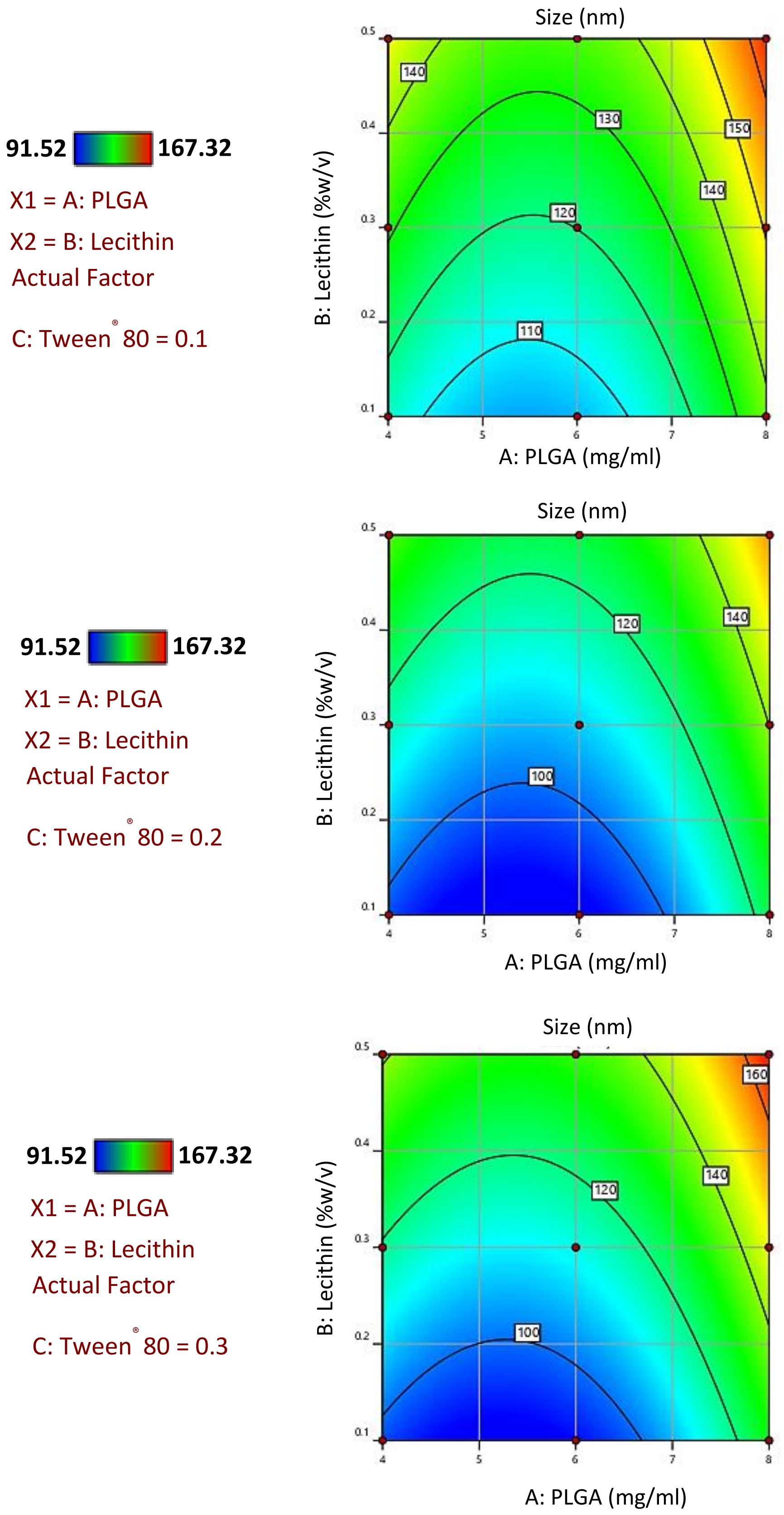

Figure S1. Effect of PLGA and lecithin concentration on PS. Contour plots illustrating the effects of three levels of PLGA and Lecithin concentrations on particle size using 3^3 factorial design at three levels of Tween® 80 concentration: 0.1 % (top), 0.2 % (middle), 0.3 % (bottom) % (w/v).

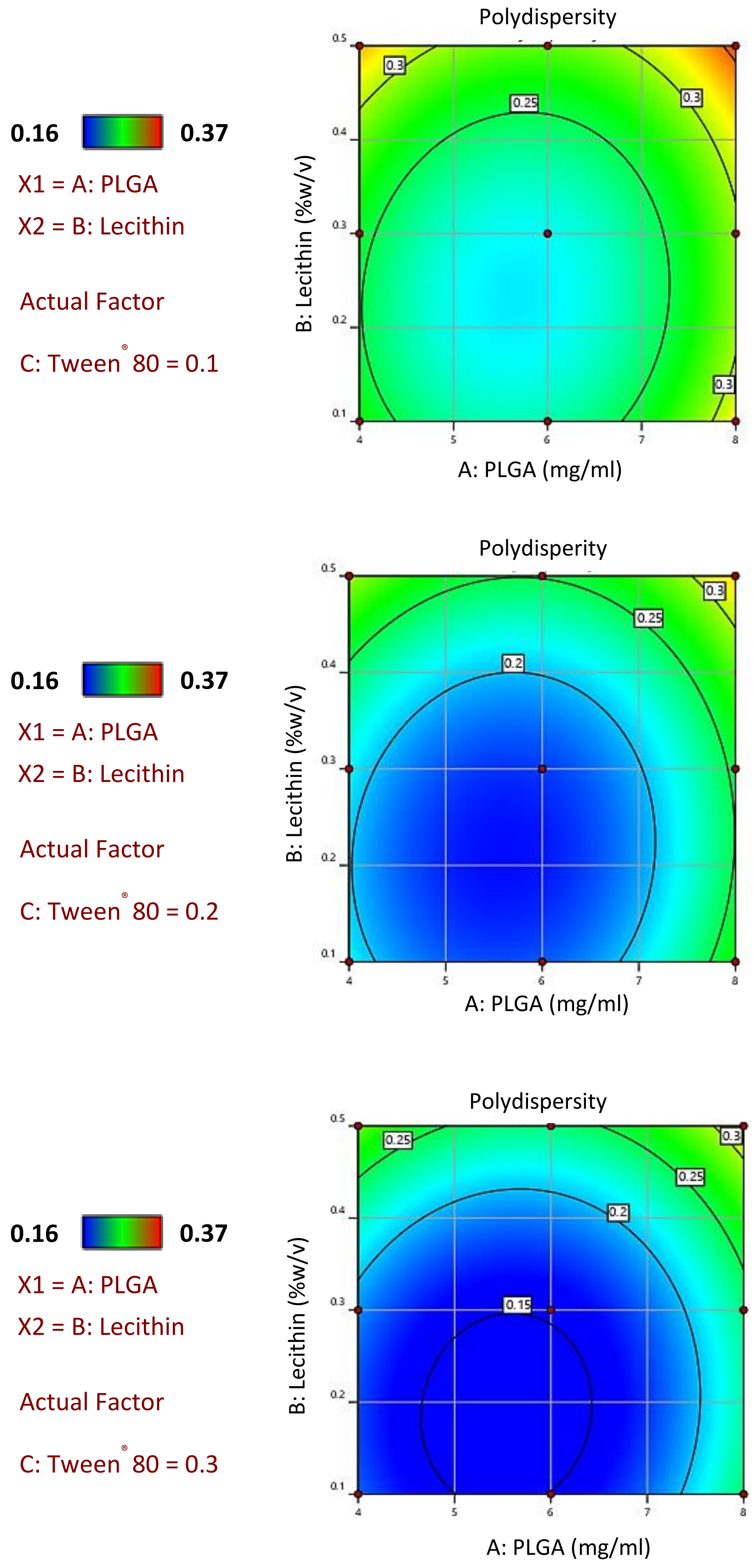

Figure S2. Effect of PLGA and lecithin concentration on PDI using 3^3 factorial design. 3D surface plots illustrating the effects of PLGA and Lecithin concentrations on PDI at three levels of Tween® 80 concentration: 0.1 % (top), 0.2 % (middle), 0.3% (bottom) (% w/v).

Table S3. ANOVA results for response surface quadratic model of PS.

Source	Sum of Squares	Df	Mean Square	F-value	p-value	
**Model**	11765.59	9	1307.29	38.29	<0.0001	**Significant**
A-PLGA	2605.22	1	2605.22	76.27	<0.0001	
B-Lecithin	5598.23	1	5598.23	163.89	<0.0001	
C-Tween® 80	269.04	1	269.04	7.88	0.0121	
AB	30.37	1	30.37	0.8891	0.3589	
AC	59.81	1	59.81	1.75	0.2033	
BC	92.57	1	92.57	2.71	0.1181	
A^2	2622.95	1	2622.95	76.79	<0.0001	
B^2	0.1350	1	0.1350	0.0040	0.9506	
C^2	487.26	1	487.26	14.26	0.0015	
Residual	580.69	17	34.16			
Cor total	12346.28	26				
**FIT STATISTICS**						
**Standard Deviation**	**Mean**	**C.V.%**	**R^2**	**Adjusted R^2**	**Predicted R^2**	**Adequate Precision**
5.84	127.30	4.59	0.9530	0.9281	0.8818	21.5850

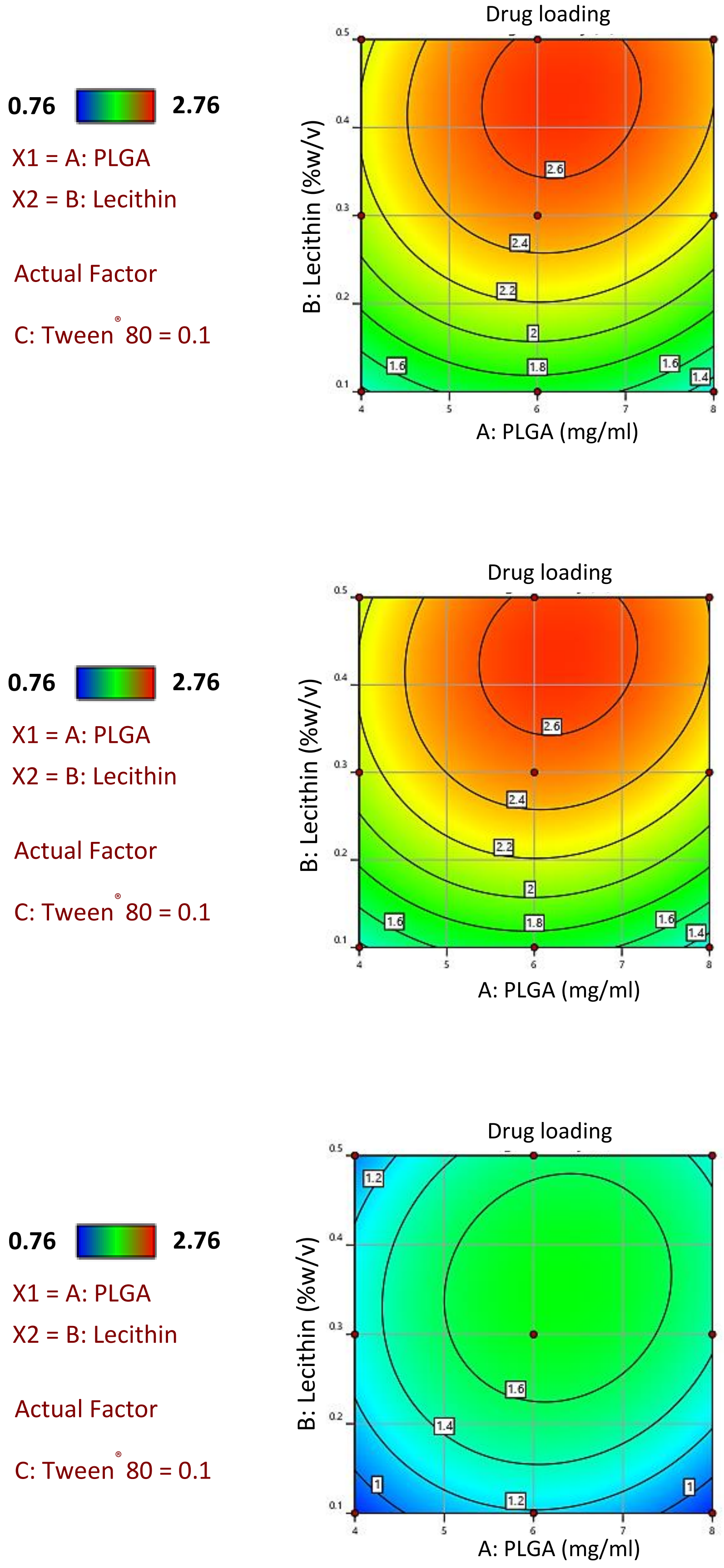

Figure S3. Effect of PLGA and lecithin concentration on DL% using 3^3 factorial design. 3D surface plots illustrating the effects of PLGA and Lecithin concentrations on DL% (Y3) at three levels of Tween® 80 concentration: 0.1 % (top), 0.2 % (middle), 0.3 % (bottom) (w/v).

Table S4. ANOVA results for response surface quadratic model of PDI.

Source	Sum of Squares	Df	Mean Square	F-value	p-value	
**Model**	0.859	9	0.0095	30.09	<0.0001	**significant**
A-PLGA	0.0084	1	0.0084	26.37	<0.0001	
B-Lecithin	0.0240	1	0.0240	75.61	<0.0001	
C-Tween® 80	0.0172	1	0.0172	54.35	<0.0001	
AB	0.0006	1	0.0006	1.85	0.1911	
AC	0.0001	1	0.0001	0.3405	0.5672	
BC	0.0010	1	0.0010	3.01	0.1009	
A^2	0.0228	1	0.028	71.94	,0.0001	
B^2	0.0110	1	0.0110	34.71	<0.0001	
C^2	0.0008	1	0.0008	2.65	0.1220	
Residual	0.0054	17	0.0003			
Cor total	0.0913	26				
**FIT STATISTICS**						
**Standard Deviation**	**Mean**	**C.V.%**	**R^2**	**Adjusted R^2**	**Predicted R^2**	**Adequate Precision**
0.0178	0.2499	7.13	0.9409	0.9097	0.8549	19.2021

Table S5. ANOVA Results for Quadratic Model of DL%.

Source	Sum of Squares	Df	Mean Square	F-value	p-value	
**Model**	6.87	9	0.7631	20.08	<0.0001	**Significant**
A-PLGA	0.0672	1	0.0672	1.77	<0.2011	
B-Lecithin	1.84	1	1.84	48.49	<0.0001	
C-Tween® 80	2.87	1	2.87	75.56	<0.0001	
AB	0.0768	1	0.0768	2.02	0.1733	
AC	0.0030	1	0.0030	0.0791	0.7819	
BC	0.2523	1	0.2523	6.64	0.0196	
A^2	0.7776	1	0.7776	20.46	0.0003	
B^2	0.7633	1	0.7633	20.08	0.0003	
C^2	0.2128	1	0.2128	5.60	0.0301	
Residual	0.6462	17	0.0380			
Cor total	7.51	26				
**FIT STATISTICS**						
**Standard deviation**	**Mean**	**C.V.%**	**R^2**	**Adjusted R^2**	**Predicted R^2**	**Adequate Precision**
0.1950	1.70	11.47	0.9140	0.8685	0.8027	15.1870

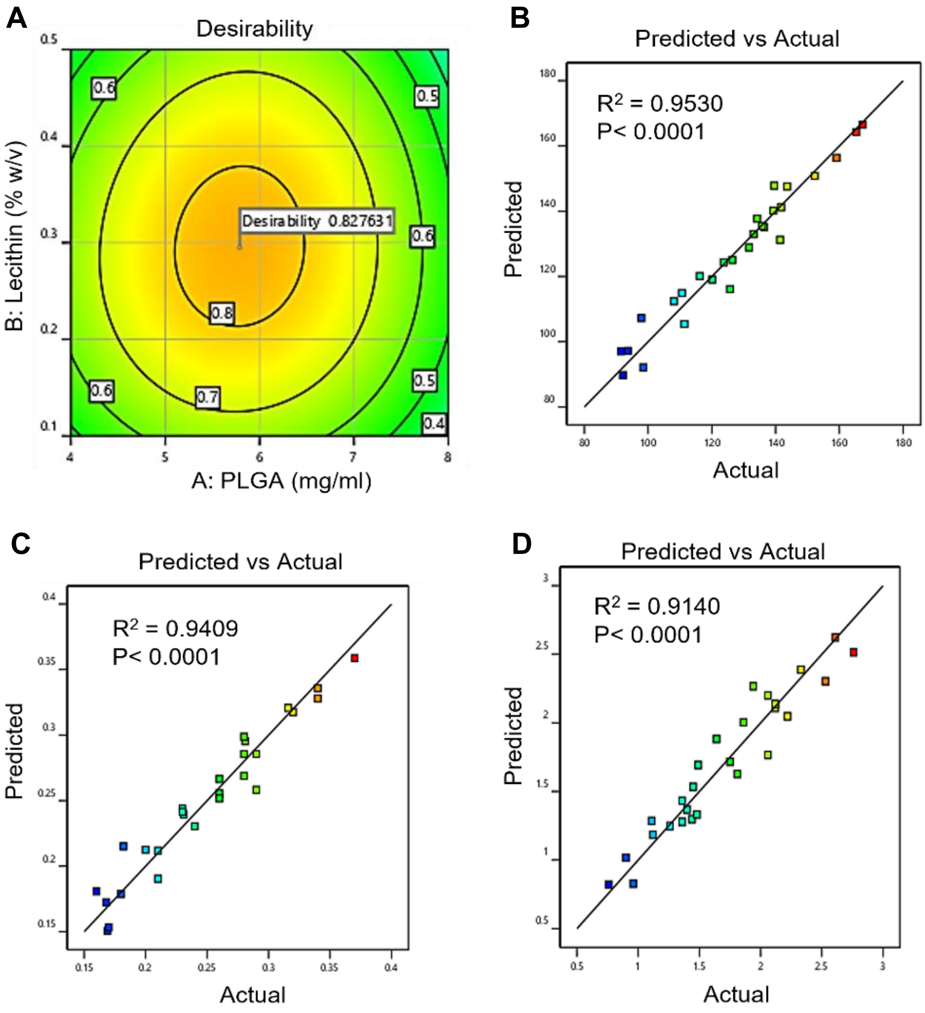

Figure S4. Desirability function and DoE model fit analysis. Contour plot showing the desirability range of independent variables for achieving optimised OT-NP (A). Scatterplots of Predicted versus experimental values modelled by multiple regression based on DoE for particle size (B), polydispersity (C) and drug loading efficiency (D). The model analysis was significant (p<0.0001) with R^2 over 90 %.

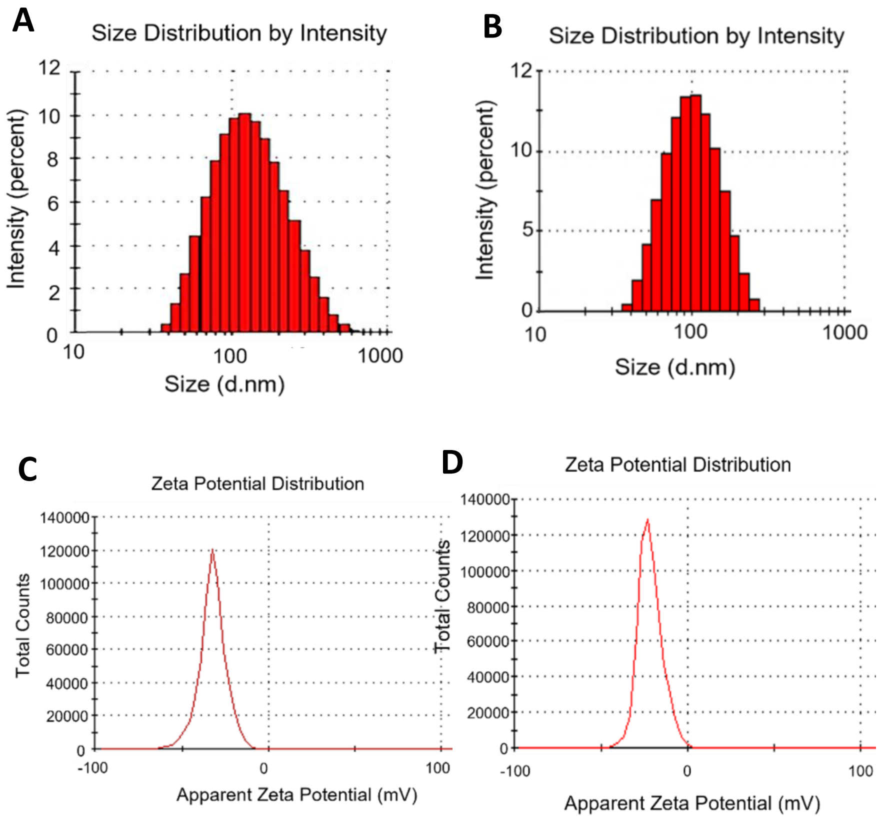

Figure S5. Representative histograms and Zeta potential graphs of OT-NP and OT-NP-PEG. Size distribution of (A) OT-NP and (B) OT-NP-PEG by intensity showing 114.8 nm and 94.9 nm, respectively, measured using dynamic light scattering. The hydrodynamic size was presented as the average value for 15 to 20 runs. Zeta potential distribution results of (C) OT-NP and (D) OT-NP-PEG showed -32.6 and -21.7 mV, respectively. Measurement was performed in triplicate using the Helmholtz–Smoluchowski equation.

Table S6. Model validation by comparing the predicted *vs* observed experimental values

	Size (Y1) (nm)[a, c]	PDI (Y2)[a, c]	Drug loading (%)[b, c]
**Predicted values**	107.70	0.172	2.38
**Experimental values**	116.30	0.180	2.76
**Validity**	92.60 %	95.5 %	115.96 %

a Measured by dynamic light scattering

b Quantified by RP-HPLC

c Expressed as mean ± SD (n=3)

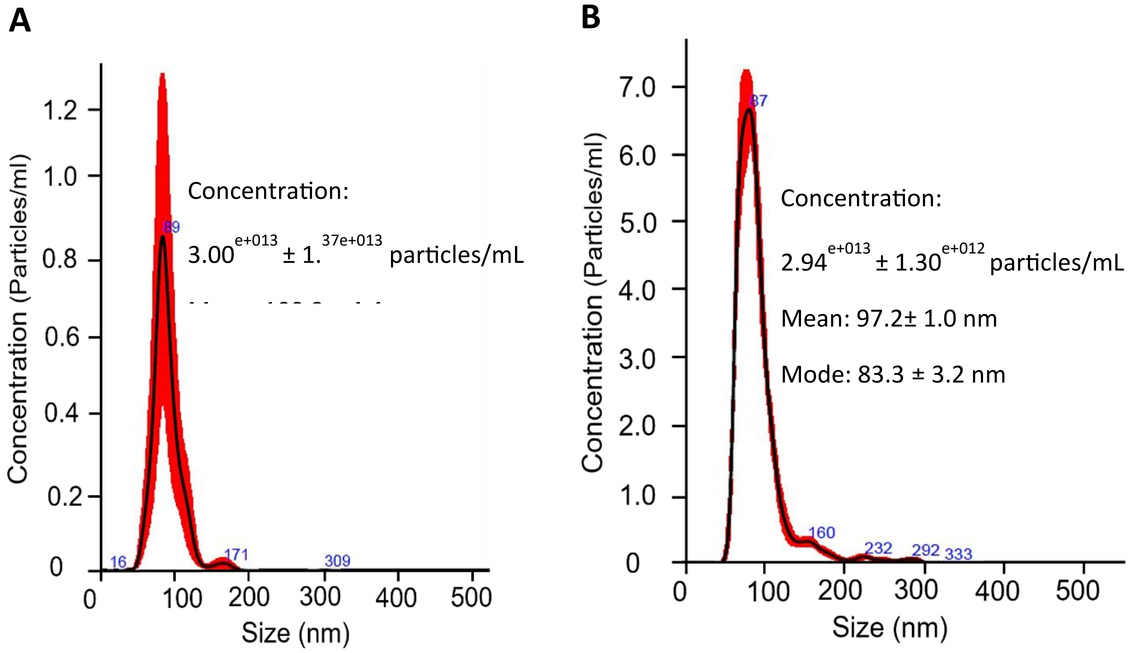

Figure S6. Characterization of OT-NP and OT-NP-PEG. Representative size distribution data of OT-NP (A) and OT-NP-PEG (B) obtained by Nanoparticle Tracking Analysis (NTA) using a NanoSight LM-10. The histograms indicate a smaller size distribution profile for OT and OT-NP-PEG.

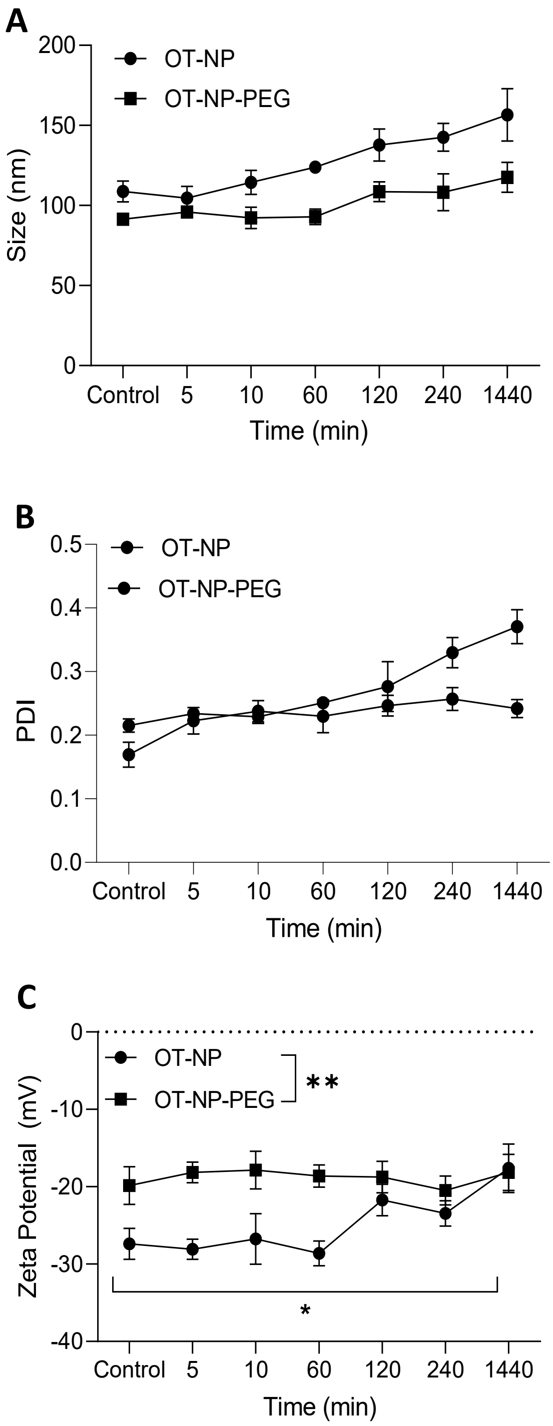

Figure S7. Effect of SNM on physicochemical properties of OT-NP and OT-NP-PEG. The 200 µL sample of 10 x concentrated OT-NP or OT-NP-PEG was mixed with 200 µL SNM in an Eppendorf tube and incubated for 24 h at 37 C. Sample was taken at different time intervals and diluted 1000 times. The hydrodynamic size (A), PDI (B), and zeta potential distribution (C) were measured using the Helmholtz–Smoluchowski equation. Measurements were done in triplicate, and data are presented as mean± SD (n=3), **P<0.01 (Unpaired Student's t-test).

Table S7. Shelf-Life Stability of OT-NP and OT-NP-PEG.

Formulation	Day	Size (nm) [a, d]	PDI [a, d]	Zeta potential (mV) [b, d]	% EE [c, d]	% DL [c, d]
OT-NP	0	113.2 ± 1.67	0.190 ± 0.023	-33.75 ± 1.7	10.39 ± 1.1	2.77 ± 0.7
	7	115.4 ± 2.50	0.237 ± 0.020	-31.18 ± 2.5		
	14	118.6 ± 2.16	0.269 ± 0.021	-22.86 ± 2.2		
	28	123.2 ± 2.18	0.2877 ± 0.03	-26.25 ± 1.7		
OT-NP-PEG	0	91.2 ± 2.64	0.210 ± 0.03	-19.56 ± 1.6	12.37 ± 1.24	3.56 ± 0.2
	7	87.3 ± 2.55	0.253 ± 0.02	-15.2 ± 2.3		
	14	94.6 ± 3.76	0.241 ± 0.04	-13.6 ± 3.2		
	28	102.2 ± 5.61	0.284 ± 0.084	-16.3 ± 2.4		

[a] Measured by dynamic light scattering
[b] Zeta potential, calculated from electrophoretic mobility measurements
[c] Calculated as encapsulated OT divided by the weight of the polymer, determined by RP-HPLC
[d] Expressed as mean ± SD (n=3)

Table S8. Fmoc solid phase peptide synthesis of OT.

Fmoc-SPPS [a]	Scale (mmole/g)	Access	% Yield (mass)	% Yield (HPLC) [b, c]
Oxytocin [1,2,4]	0.05	6	87	84
	0.0125	6	94.91	90.67

[a] TentaGel® resin as a solid support (capacity 0.2 mmole/g)
[b] Characterized by RP-HPLC and LC-MS
[c] Purified by SPE column

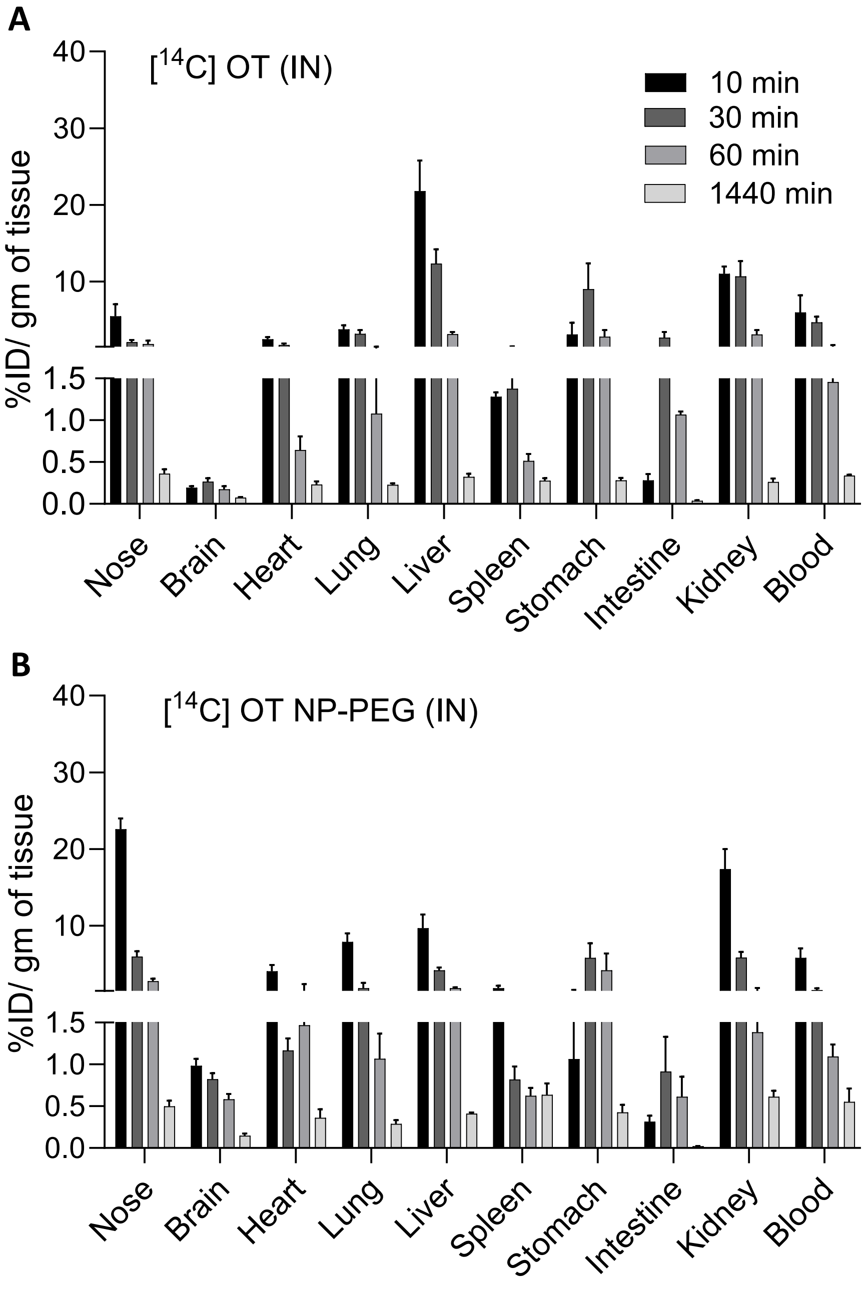

Figure S8. *In vivo* whole organ biodistribution of [^{14}C] OT and [^{14}C] OT-NP-PEG after IN administration. Mice were administered IN with 20 μL (10 μL each nostril) [^{14}C] OT or [^{14}C] OT-NP-PEG in PBS under inhalation. At the terminal time points (10, 30, 60 or 1440 min), mice were euthanized under terminal anesthesia and blood was withdrawn from the inferior vena cava. Major organs were collected and processed for LSC. The % ID per g of tissues is presented for (A) soluble OT or (B) [^{14}C] [^{14}C] OT-NP-PEG. Data are expressed as mean ± SEM, n = 3.

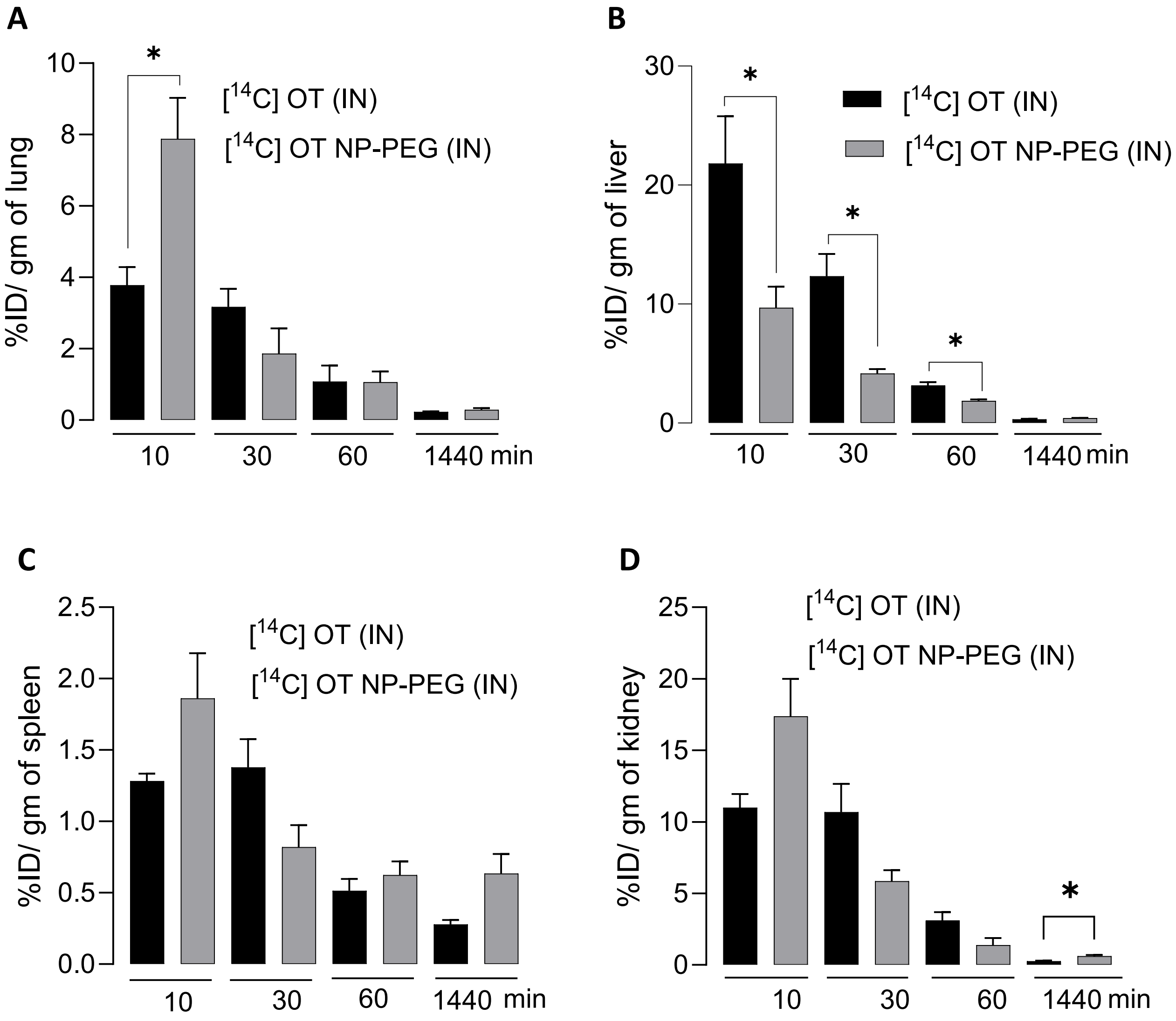

Figure S9. Uptake of [^{14}C] OT and [^{14}C] OT-NP-PEG in lung, liver, spleen and kidneys after IN administration. Mice were administered IN with 20 µl (10 µl each nostril) [^{14}C] OT or [^{14}C] OT-NP-PEG in PBS under inhalation anesthesia. At the terminal time points (10, 30, 60 or 1440 min), mice were euthanized under terminal anesthesia, and blood was withdrawn from the inferior vena cava. Major organs were collected and processed for LSC. The % ID per g tissues of (A) lung, (B) liver, (A) spleen, and (D) kidneys is presented for soluble [^{14}C OT or [^{14}C OT-NP-PEG. Data are expressed as mean ± SEM, *n* = 3. **p < 0.05, **p < 0.01* (One-way ANOVA followed by Tukey's multiple comparison test).

Table S9. Fmoc-solid phase peptide synthesis of [^{14}C] OT.

Fmoc-SPPS [a]	[^{14}C] OT	
Scale (mmole)	0.01	0.05
Excess	10	5
% Yield (mass)	89.0	94.6
% Yield (RP-HPLC) [b,c]	60	74.44
% Radiochemical yield (HPLC) [b]	9.48	53.35
Specific radioactivity (µCi/mg) [d]	0.79	4.45

[a] TentaGel® resin as solid support (capacity 0.2 mmole/g)
[b] Characterized by RP-HPLC
[c] Purified by SPE column
[d] Quantified by LSC

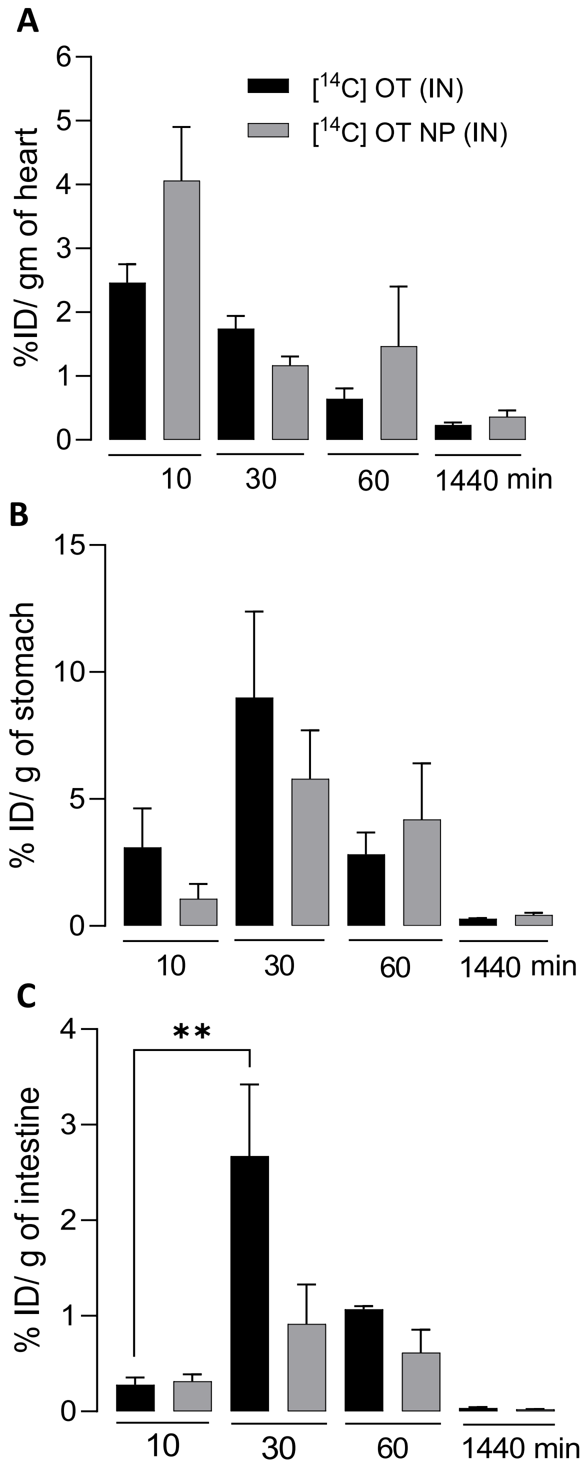

Figure S10. Uptake of [14C] OT and [14C] OT-NP-PEG in heart, stomach and intestine after IN administration. Mice were administered IN with 20 µL (10 µL each nostril) [14C] OT or [14C] OT-NP in PBS under inhalation anesthesia. At the terminal time points (10, 30, 60 or 1440 min), mice were euthanized under terminal anesthesia, and blood was withdrawn from the inferior vena cava. Major organs were collected and processed LSC. The % ID per g tissues of heart (A), stomach (B) or intestine (C) is presented for soluble [14C] OT or [14C] OT-NP-PEG. Data are expressed as mean ± SEM, n = 3. $^{**}p < 0.01$ (One-way ANOVA followed by Tukey's multiple comparison test.

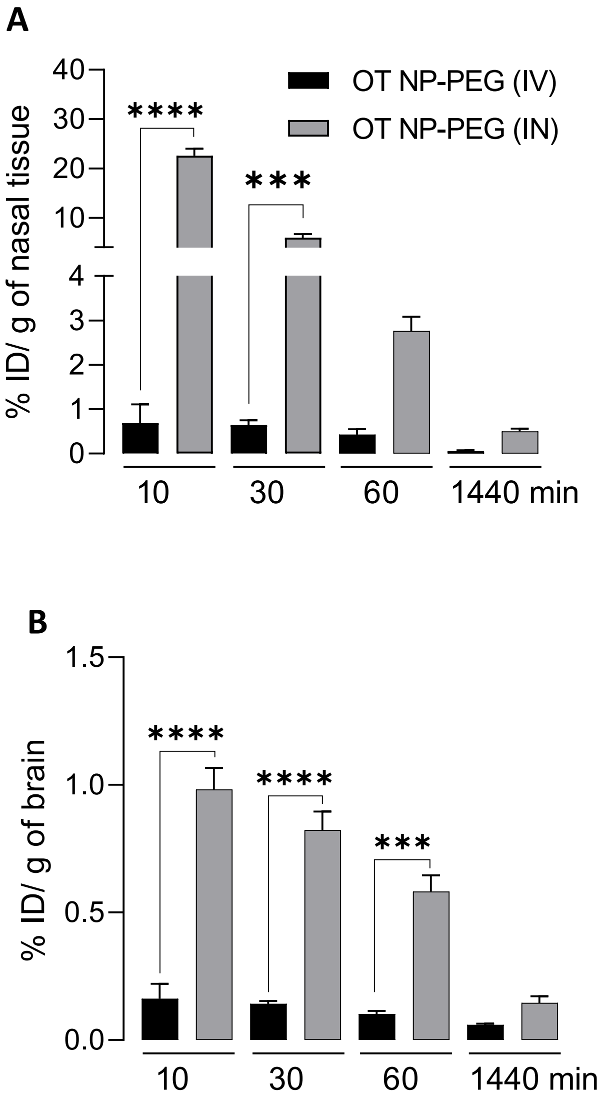

Figure S11. Uptake of OT-NP-PEG in nasal tissues and brain after IN and IV administration. Mice were administered IV with 100 µl or IN with 20µl (10µl each nostril) OT or OT-NP in PBS under inhalation anesthesia. At the terminal time point (10, 30, 60 or 1440 min), mice were euthanized under terminal anesthesia and blood was withdrawn from the inferior vena cava. Major organs were collected and processed for liquid scintillation counting. The % ID per gram tissues of nasal tissues (A), and brain (B) is presented for soluble OT or OT-NP-PEG. Data are expressed as mean ± SEM, n = 3.; ****$p < 0.0001$* (One-way ANOVA followed by Tukey's multiple comparison test).

Table S10. Fluorescence labelling of OT-NP and OT-NP-PEG.

Formulation	Size (nm) [a, d]	PDI [a, d]	ZP (mV) [b, d]	% Labelling efficiency [c, d]
OT-NP (DiI)	114.25 ± 3.1	0.216 ± 0.05	-25.6 ± 4.2	84.22 ± 5.31
OT-NP (blank)	105.33 ± 6.4	0.197 ± 0.03	-31.3 ± 3.3	--
OT-NP-PEG (DiI)	107.61 ± 2.4	0.204 ± 0.07	-14.4 ± 2.7	78.34 ± 7.1
OT-NP-PEG (blank)	91.67 ± 4.6	0.188 ± 0.04	-21.2 ± 3.7	--

[a] Measured by dynamic light scattering
[b] Zeta potential, calculated by electrophoretic mobility
[c] Calculated as encapsulated DiI divided by total dye added determined from fluorescent intensities
[d] Expressed as mean ± SD (n=3)

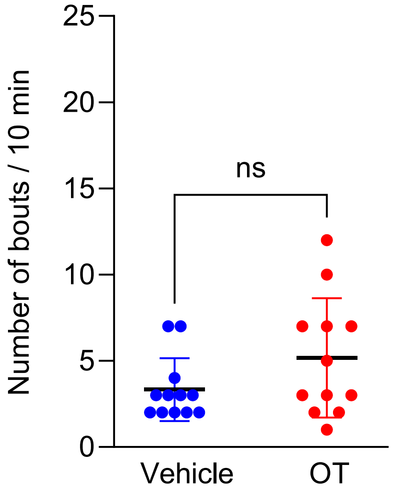

Figure S12. Effect of Vehicle and free OT on self-grooming behaviors in mice after IP administration. Figure showing the effect of IP Vehicle (10 mL/kg, sterile PBS) and OT (50 mg/kg) on the frequency and duration of self-grooming (number of bouts) behaviour, scored for 10 min after 5 min habituation period. The dosing and analyses were carried out in a blinded manner. Data are expressed as mean ± SEM, n = 12.

Bibliography

1. Ahmed, O.A., et al., *Application of nanopharmaceutics for flibanserin brain delivery augmentation via the nasal route.* Nanomaterials, 2020. **10**(7): p. 1270.
2. Masiuk, T., P. Kadakia, and Z. Wang, *Development of a physiologically relevant dripping analytical method using simulated nasal mucus for nasal spray formulation analysis.* Journal of pharmaceutical analysis, 2016. **6**(5): p. 283-291.
3. Türeli, N.G., et al., *Ciprofloxacin-loaded PLGA nanoparticles against cystic fibrosis P. aeruginosa lung infections.* European Journal of Pharmaceutics and Biopharmaceutics, 2017. **117**: p. 363-371.
4. Lababidi, N., et al., *Microfluidics as tool to prepare size-tunable PLGA nanoparticles with high curcumin encapsulation for efficient mucus penetration.* Beilstein Journal of Nanotechnology, 2019. **10**(1): p. 2280-2293.
5. Yang, Q. and S.K. Lai, *Anti-PEG immunity: emergence, characteristics, and unaddressed questions.* Wiley Interdisciplinary Reviews: Nanomedicine and Nanobiotechnology, 2015. **7**(5): p. 655-677.
